# Selective emotion regulation in creative art production: Psychophysiological reactivity during painting reduces anxiety

**DOI:** 10.1016/j.isci.2025.112543

**Published:** 2025-04-28

**Authors:** Lucas Bellaiche, Kayla Lihardo, Chloe Williams, Jill Chaffee, Kevin S. LaBar, Paul Seli

**Affiliations:** 1 Department of Psychology and Neuroscience, Duke University, Durham, NC, USA

**Keywords:** Psychology

## Abstract

Across the literatures of aesthetics, philosophy, and psychology, art has long been revered as a powerful means to enhance mental well-being—a perspective that has been integrated into clinical practices worldwide. While some empirical research supports the emotional benefits of art production, such work often captures non-creativity factors (e.g., physical movement and social interaction), leaving the contribution of creative expression on psychophysiological outcomes unclear. To address this issue, we conducted a pre-registered, multi-modal, repeated-measures study wherein participants completed both a painting task and a non-creative but active control task. Our findings demonstrate that, above and beyond the non-creativity processes shared with the control task, painting selectively reduces anxiety, and that greater cognitive engagement and physiological reactivity characterize this reduction. These findings highlight the multi-modal determinants of emotional improvement during artistic production, providing empirical support for the therapeutic benefits of art-making specific to the regulation of anxiety.

## Highlights

• Creating a painting lowers anxiety more than a non-creative control task
• Anxiety reduction is smaller in both tasks for those who feel greater boredom
• Painting yields higher physiological reactivity, especially in more-creative people
• During painting, higher physiological reactivity predicts greater anxiety reduction

## Graphical abstract

**Figure:**
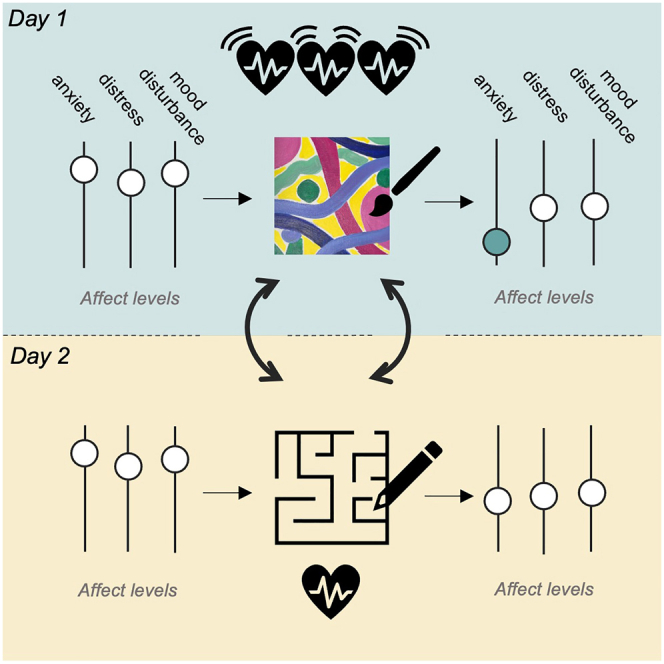

## Introduction

The role of emotions has been a topic of great emphasis within the domain of aesthetics. Many believe that emotional responses––ranging from feelings of basic preference^1^^,^^2^ to those of transcendental awe^3^^,^^4^––are an essential feature of an individual’s personal encounters with art (e.g., Chatterjee & Vartanian^5^; Leder et al.^6^). These emotional responses extend beyond personal experiences, influencing broader societal interactions by fostering socio-cultural bonds, enhancing artist-audience understanding, reinforcing cultural values, and training emotion-related operations for real-world applications.^7^

While a large proportion of the psychoaesthetics literature focuses on emotional mechanisms among art *consumers* (drawing from Kant’s original proposition of “aesthetic emotions”^8^), rich emotional stimulation can also occur within art *producers*. In fact, the emotional dynamics experienced among individuals as they create art can be leveraged toward mood repair and emotion regulation (ER).^9^^,^^10^^,^^11^^,^^12^^,^^13^^,^^14^^,^^15^^,^^16^ In the present study, we bring into focus the creative expression involved during art production and its influence on psychophysiological outcomes of ER.

The study of art enjoys a rich and diverse literature, spanning centuries of examinations across the disciplines of philosophy, psychology, and the fine arts. Despite numerous approaches to understanding art, scholars have failed to agree on a fixed definition of “art,” as there does not appear to be any one essential feature that unites all art, unlike, say, prime numbers.^17^ Indeed, art is a complex social construct that comprises diverse modalities, including dancing, painting, poetry, and music, to name a few. The scientific field of psychoaesthetics investigates our psychological interactions with these many domains, yet much of this research fails to present a central operationalization of “art” that both encompasses across modalities and also distinguishes art from “non-art”: an issue highlighted in recent years within the psychoaesthetics literature.^13^^,^^15^^,^^17^ In this paper, although we utilize the particular modality of abstract painting, we rely on the following working definition of art more broadly: the physical expression of creative cognitive processes via one of many possible modalities (e.g., visual art, music, and poetry).^18^ Indeed, art is a clear and intuitive example of creative expression, as it inherently involves the imaginative and innovative creation of new and original works (i.e., creativity)^18^^,^^19^ (see also Diedrich et al.^20^; Ivcevic^21^). This paper also refers to those who produce this art––regardless of expertise in the domain––as artists. Importantly, while our operationalization of art permits us to more clearly investigate the foundational processes involved in the creation of art like creative expression, it should be noted that the psychological factors involved in art production that correspond with such creative expression remain poorly understood. This paper aims to investigate one such factor: emotion.

### The affective power of art production

#### Potential mechanisms

The investigation into the specific affective properties of art production has yielded prolific lines of research. One such line refers to clinical-specific studies of art therapy, where clinicians leverage engagement with art––either producing or consuming––as a means of psychological healing, particularly emotional relief, in patients (e.g., watercoloring in cancer patients^9^; writing and drama in those suffering from chronic somatic illnesses^22^).^23^ Similarly, researchers have also examined the benefits of art production in non-clinical samples more broadly, an approach more reflective of the current study’s methodology (e.g., Abbott et al.^24^; Kaimal et al.^25^; Sandmire et al.^26^; Sandmire et al.^27^) (although these two lines of work leverage unique samples, our literature review consolidates both due to their similarities in study designs and aims). Across these spheres of work, some studies have attempted to elucidate the specific ER mechanisms underlying art production. One explanation is that individuals actively employ different cognitive strategies while producing art, such as reflecting on current emotions or intentionally distracting themselves from external stressors.^10^^,^^28^ Another explanation for ER during art production is the concept of *flow*: an immersive and transient state of concentrated engagement with a task.^29^ Although this mode of thought typically occurs automatically and without conscious effort,^30^ flow is often associated with subjective feelings of positive affect^31^ and, as Nakamura and Csikszentmihalyi^32^ (p. 102) have proposed, it “serves as a buffer against adversity and prevents pathology.” Artistic production reliably evokes such flow states across various modalities, such as music^33^ and visual art.^34^^,^^35^ These flow states, in turn, predict mood repair and ER.^34^^,^^36^^,^^37^^,^^38^^,^^39^

When considering flow in the context of ER, it is also important to consider the experience of boredom. In fact, flow was originally defined in relation to boredom^29^: whereas flow occurs when skill and demand are optimally balanced, boredom occurs when there is an excess of individual skill compared to environmental demand.^40^ Thus, it is thought that boredom emerges opposite to flow,^41^^,^^42^ and studies examining both flow and boredom in tandem have shown a negative correlation between these two states.^43^^,^^44^ Given this theoretical and empirical grounding, it is reasonable to hypothesize that boredom would have the opposite relation to ER compared to flow, potentially leading to negative affect and reduced ER. Indeed, work has shown boredom to be predictive of difficulties in implementing successful ER^45^ and also predictive of maladaptive regulatory strategies, including emotional eating,^46^ negative self-evaluations,^47^ substance use,^48^ and even self-injury.^49^

#### Methodological concerns

##### Control conditions

Despite these advances in understanding the potential mechanisms underlying ER during art production, significant methodological issues remain. For instance, perhaps the most prominent concern is the vague operationalization of “art,” as aforementioned.^17^ As echoed by recent calls across cognitive and clinical spheres,^13^^,^^15^^,^^17^ by explicitly specifying features of art of interest, researchers can actively investigate which, if any, aspects of artistic production might contribute positively to ER and well-being. Of course, art-making is a multifaceted process involving various faculties. Common variables in art production—each potentially contributing to some emotional benefit—include creative expression, motor activity, social interaction, and contextual factors like being in a new environment (see Uttley et al.^15^). Therefore, it is crucial for researchers to use appropriate control conditions to isolate the effects of the target variable. However, prior research has struggled with methodological issues related to control conditions, leading to potentially confounding influences from extraneous variables.^13^^,^^15^^,^^17^ For example, Bozcuk et al.^9^ non-randomly assigned 48 cancer patients to an art therapy intervention and 24 to a no-task control. While the intervention group showed significant improvements in quality of life and depression, these benefits could be due to general engagement in a leisure activity rather than the specific effects of creating art. Similar concerns apply to other studies that lack an active control task (e.g., Sandmire et al.^26^; see Sharp & Hewitt^14^) or lack comparative conditions more generally (e.g., Forzoni et al.^50^; Kaimal et al.^25^).

One well-accepted component of art that could be isolated with the aid of control conditions is creative expression, the variable we consider to be most inherent to art making. Crucially, theoretical accounts also posit that it is specifically creativity that regulates emotions and enhances well-being.^51^ Mechanistically, approaching an artistic problem encourages divergent cognitive simulation for possible opportunities and interpretations (see Addis et al.^52^) and yields feelings of positive reward.^53^ However, empirical evidence on whether creative expression in art supports ER is mixed, largely due to the lack of studies comparing art to non-creative tasks. For instance, Abbott et al.^24^ found that among their non-clinical sample of undergraduate students, representational painting reduced stress more than geographical map puzzles, attributing this to creativity and positive focus. Conversely, though, Volpe et al.^54^ found no significant difference in quality-of-life improvements between creative dance interventions and traditional physiotherapy for Parkinson’s patients, which brings into question the unique role of creative expression in well-being. Collectively, while many studies demonstrate a positive impact of artistic engagement on well-being, claims regarding the healing aspects of creativity are often limited by inadequate or lacking comparative conditions.

##### Psychological versus physiological data

Another consideration of many empirical investigations of art production is the reliance on self-reported data as metrics of ER. While these measures are valuable in capturing the subjective nature of well-being, their validity is inherently limited by aspects like demand characteristics and how questions are phrased (see Schwarz^55^). Furthermore, these self-report questionnaires often capture widely varying outcome measures (e.g., mood, anxiety, stress, well-being, self-esteem, social skills, and domain-specific aspects like motor skills in movement disorders^13^^,^^14^), which ultimately undermines the ability to make generalizable claims about the emotional benefits of art production across studies. Accordingly, psychoaesthetic researchers have called for a shift toward multi-method studies that incorporate physiological data to comprehensively examine the impact of art engagement on both the mind and body^16^^,^^17^ (see also Shields^56^ for stress-related paradigms).

One common approach to examining physiological reactivity is to measure features of heart rate (HR). Broadly conceptualized as the change in activity of HR during a task,^57^ HR reactivity captures important signals relating to cardiovascular dynamics and its interactions with cognitive states like anxiety. Several methods have been proposed for capturing HR reactivity. For example, in Deutz et al.,^58^ HR reactivity was operationalized as the difference in HR between two set time points: the end minus the beginning of a stress induction block. Alternatively, Turner et al.^57^ suggest to “subtract the baseline level from the level of activity during the stress” (p. 3), essentially capturing a peak-to-trough (or maximum-minus-minimum) measurement.

Beyond simple subtractions of HR values like end-minus-beginning and peak-to-trough, some clinicians have suggested that volatility is another helpful measure of reactivity.^59^ That is, by calculating variation-related statistics like standard deviation over a window of continuous heart rate during some task, one can gain perspective on novel dynamics of HR, and Grogan et al.^60^ even hypothesize that HR volatility “is a surrogate for autonomic nervous system dysfunction” (p. 547). Lastly, another measurement of HR reactivity is simply the average HR produced across the duration of a given task. While this may be less sensitive to volatility of HR, it nonetheless captures the central tendency of an individual’s physiological dynamics and could complement the aforementioned variables well if measured altogether.

##### (Non-)generalizable samples

Lastly, as evidenced by our literature review, much of the research in this field is labeled as art therapy, and specifically targets interventions in clinical samples (i.e., individuals with diagnosed physical or psychological conditions). This focus is important because it highlights the potential cognitive and affective benefits in clinical populations and provides an avenue for a broad, cost-effective, and generally fun treatment for individuals who could benefit. Nonetheless, as research has shown across student populations^61^—including undergraduates^24^^,^^62^^,^^63^ and nurses^64^—art engagement could also benefit individuals who may not have a diagnosed condition but who nonetheless face daily stressors, negative affect, and feelings of anxiety and stress. Additionally, short-term interventions for these non-clinical samples (in contrast to multi-week clinical therapies) have yielded significant increases in positive emotions (and decreases in negative ones) as in a one-session mindfulness-based doodle therapy.^65^ Thus, art production can assist individuals across the mental and physical health spectrums on relatively short timescales, although much of the prior work has been targeted to specific clinical conditions which subsequently hinders the generalizability of such work.

### The present study

The present study sought to examine the multi-modal magnitude, correlates, and determinants of improvement in mental health stemming from the creative expression that is inherent in art making. We used a randomized, cross-over, within-subjects design entailing an abstract painting condition and an active, non-creative control condition matched for social environment, motor movement, and duration. The participants––who were recruited from the surrounding campus without clinical inclusion or exclusion criteria and included undergraduate students, graduate students, and staff––completed both conditions on separate days in a randomized order to maximize statistical power and emphasize within-person difference. We aimed to collect a wide range of behavioral data, including pre- and post-condition difference scores across three common indices of state-level mental health: mood disturbance, anxiety, and distress. Self-report data were combined with continuous monitoring of HR data concurrent with each condition to capture physiological reactivity via wearable devices. We also administered measures assessing potential mediating variables as suggested by the broader creativity literature (e.g., flow), as well as trait-level differences in mental health and in real-world creative activities and achievements.

#### Abstract painting

While a diverse array of modalities has been investigated in art production studies, until recently, one notable exclusion has been abstract painting. Our research has demonstrated that abstract painting can serve as a feasible index of individual creative ability.^66^ Abstract painting also enjoys the added methodological benefit of containing no representational figures. Indeed, the capacity to adequately paint a specific figure (as in representational art) may invoke several confounds, including feeling an added level of pride or shame based on how well the figure mirrors the real-life object (see Forkosh and Drake,^34^ p. 80). Additionally, the sheer ability to accurately paint a figure and derive these emotions may be attainable only through training and expertise, which subsequently limits the accessibility of art-production interventions. Lastly, these drawn figures themselves may contain unintended emotional associations with memories that could alter ER processes; for instance, an individual may begin to paint a tree in an attempt to reflect the calming experience of nature, only to remember a certain negative memory with a tree in that individual’s past. Thus, our design’s use of abstract painting satisfies calls by researchers like Forkosh and Drake^34^ to diversify art production paradigms to non-representational domains, while also circumventing concerns regarding expertise and emotional confounds given the free creative expression without representational figures.

#### Extensions from prior relevant work

While numerous studies have reported that art production can improve mental health, we sought to build on this research to provide a more accurate and holistic understanding of the role of ER during art production in non-clinical samples. In the following text, through a review of key papers, we outline important methodological and conceptual issues in the literature and describe how our work addresses these gaps.

First, as mentioned, a critical limitation in the literature is the lack of robust control conditions. Often, studies do not include conditions to compare against their art-making experimental one. If they do include multiple conditions, they may not be as appropriately active as art: the control condition of Sandmire et al.,^26^ for instance, involved no task, thus potentially attributing emotional benefits during art-making to general task engagement. One notable study with appropriate control conditions was Abbott et al.,^24^ who utilized an active non-art condition to control for such confounding variables in demonstrating anxiety reductions; we follow closely from them by using an active but non-artistic maze-completion task as our control condition. However, due to their between-subjects design (as in most art-making experiments), the role of individual variability in affective improvements during art-making remains a significant unknown: our second major point. Thus, we further extend on their paradigm by utilizing a within-subjects crossover design to maximize statistical power and leverage within-individual variance, permitting us to compare how the same individual responds to art-making versus the control task.

Third, many studies have relied solely on self-reported affective improvements, overlooking physiological measures of mental health. To address this, Kaimal et al.^25^ incorporated cortisol data to capture an alternate measure of anxiety; we expand on this work by capturing cardiac measures of HR and complementing these physiological measurements with validated questionnaires of self-reported mental health. This more-comprehensive design mirrors that of Sandmire et al.,^27^ who likewise measured physiological data alongside self-reported anxiety using a within-subjects design. However, we also administered general mood and cognitive distress measures in addition to anxiety, and recruited a larger sample size for power purposes.

Fourth, art varies by modality. So, while Ashlock et al.^62^ found that various visual artistic activities can improve mental health among undergraduate students, we extend this research to the under-researched visual domain of abstract painting, which can have methodological benefits as reported aforementioned.

Lastly, while experimental (versus control) conditions can elucidate key processes of note (like creative expression), related variables have been implicated as driving mechanisms behind such processes. Thus, we explored whether measures of task engagement would mediate the therapeutic effect of creative expression given work emphasizing the regulatory effects of flow and related variables during artistic production.^34^^,^^36^^,^^37^

#### Hypotheses

Through these collective extensions of prior work, we aimed to elucidate the impact of creative art production on ER. Based on an extensive literature review, we hypothesized in our pre-registration that painting would significantly reduce stress and improve mood, and we expected this improvement from painting to be significantly greater than the improvement from the non-art control condition due to the unique role of creativity. We further anticipated that physiological data—specifically HR—would correspond to self-reported improvements in anxiety. However, we did not include a directional hypothesis for this correlation due to what has been referred to as the “meditation paradox”^67^: although relaxation is typically associated with decreased HR, certain forms of meditative practice have been shown to increase cardiac activity despite subjective feelings of calm, particularly when the methods are cognitively demanding.^68^ On the one hand, art production may induce a deeply relaxing, low-arousal state, leading to reductions in heart rate. On the other, it may function more like these cognitively active forms of emotion regulation, in which increased physiological arousal co-occurs with reduced anxiety (see Sandmire et al.^27^). In this sense, art-making may resemble relaxed emotional states elicited during physically engaging activities such as exercise.^69^^,^^70^

## Results

### Self-report analyses

#### Primary analysis: The effect of condition on pre- versus post-condition affect ratings

**Figure 1:**
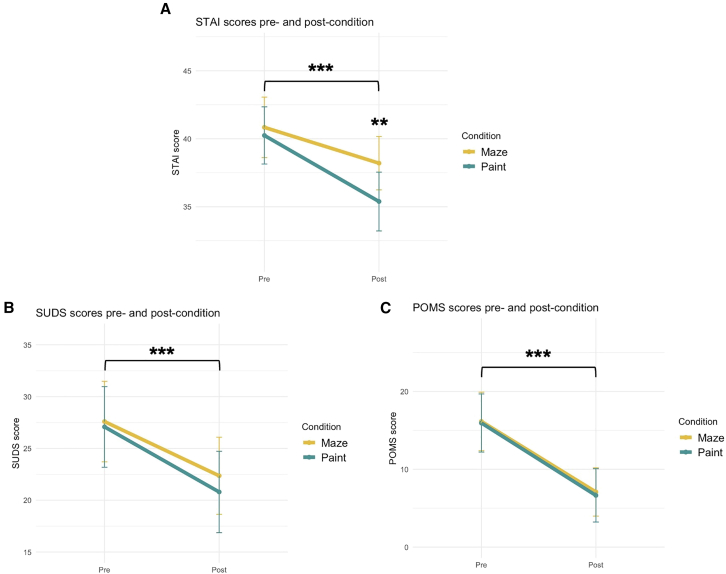
Differences in affect before and after the painting and non-creative control conditions (A) A significant main effect of Time (pre, post; *p* < 0.001) was moderated by Condition such that painting reduced STAI scores more than mazes (*p* = 0.018), leading to a significant difference in post-condition scores between conditions (*p* = 0.009). (B and C) Only a significant main effect of Time (*p*’s < 0.001) emerged for SUDS and POMS, with no support that conditions differentially reduce distress and mood disturbance, respectively. Data are represented as mean ± 95% confidence intervals. ∗∗∗*p* < 0.001 and ∗∗*p* < 0.01. See also Figures S5–S7.

Our primary research question concerned whether painting, compared to the control condition of maze completion, produced greater affective benefits as measured by the STAI (anxiety), POMS (mood disturbance), and SUDS (distress) questionnaires. A 2 (Condition: paint, maze) × 2 (Time: pre-, post-condition) repeated-measures ANOVA with STAI scores as the dependent variable revealed a significant main effect of Time, *F*(1, 98) = 38.53, *p* < 0.001, $\eta_{p}^{2}$ = 0.282, and a significant main effect of Condition, *F*(1, 98) = 3.96, *p* = 0.049, $\eta_{p}^{2}$ = 0.239. Crucially, these main effects were moderated by a significant interaction between Time and Condition, *F*(1, 98) = 5.78, *p* = 0.018, $\eta_{p}^{2}$ = 0.056. Post-hoc contrasts revealed a significant decrease in STAI scores for each condition between the time points (paint: *t*(98) = −6.741, *p* < 0.001, *b* = −4.86; maze: *t*(98) = −3.304, *p* = 0.001, *b* = −2.64), but significantly greater reductions for painting than mazes, *t*(98) = −2.404, *p* = 0.018, *b* = −2.22. The post-condition STAI scores were significantly lower for painting than mazes, *t*(98) = −2.662, *p* = 0.009, *b* = −2.818 (Figure 1A). A supplementary analysis on effect duration revealed that this anxiety reduction specific to painting did not extend into the next day (Figure S7).

Next, a similar 2 × 2 repeated-measures ANOVA was conducted for SUDS scores (distress) as the dependent variable, finding a significant main effect of Time, *F*(1, 98) = 52.73, *p* < 0.001, $\eta_{p}^{2}$ = 0.350, such that post-condition SUDS scores were significantly lower than pre-condition scores, *t*(98) = −7.262, *p* < 0.001, *b* = −5.76. However, no significant main effect was found for Condition, *F*(1, 98) = 0.47, *p* = 0.497, $\eta_{p}^{2}$ = 0.005. Additionally, no significant interaction was found between Time and Condition, *F*(1, 98) = 0.44, *p* = 0.507, $\eta_{p}^{2}$ = 0.005. Lastly, another 2 × 2 repeated-measures ANOVA was done with POMS scores (mood disturbance) as the dependent variable, again finding a significant main effect of Time, *F*(1, 98) = 92.72, *p* < 0.001, $\eta_{p}^{2}$ = 0.486, such that post-condition POMS scores were significantly lower than pre-condition scores, *t*(98) = −9.629, *p* < 0.001, *b* = −9.18. Like for SUDS, no significant main effect was found for Condition, *F*(1, 98) = 0.04, *p* = 0.834, $\eta_{p}^{2}$ < 0.001. Additionally, no significant interaction was found between Time and Condition, *F*(1, 98) = 0.02, *p* = 0.895, $\eta_{p}^{2}$ < 0.001 (all patterns of results remained the same when analyses were repeated on positive and negative subsets of items from the POMS (see Figure S6)). A supplementary analysis on effect duration revealed no extended effect into the next day for SUDS scores, and a small extended effect (across conditions) into the next day for POMS scores (Figure S7).

All significant ANOVA main effects and interactions persisted when including condition order as a between-subjects variable (see supplemental information). We also sought to examine if recent (within the previous 24 h) exercise, caffeine intake, and/or use of substances (medication, alcohol, other) moderated the aforementioned effects, as listed in our pre-registration. Three repeated-measures ANCOVAs were carried out, each with STAI, SUDS, and POMS as dependent variables, and exercise, caffeine, and substance use as covariates. All prior significant effects from the original ANOVAs remained significant. Full models can be found in the supplemental information. Overall, these results suggest painting to benefit state-level mood across all measures, but only for STAI did painting benefit significantly more than mazes did (Figure 1). Given the lack of significant Condition × Time effects––which was our primary research question––for SUDS and POMS, we henceforth only report additional analyses relating to STAI scores.

#### Trait-level moderation

Given the significant effect of Condition on anxiety reduction, we sought to determine whether certain trait-level variables moderated the anxiety benefits derived specifically from painting. This analysis aimed to examine the accessibility of emotional benefits from art production by investigating whether certain individuals experience more affective benefits from creative expression than others. To this end, we assessed trait mental health levels and creativity experience as potential moderating variables, using aggregate measures of Psychopathology and General and Visual Creativity derived from trait-level questionnaires (see STAR Methods). At the request of a reviewer, we also explored moderating roles of additional exploratory variables of potential interest, including trait-level openness to experience. All analyses (which can be found in the supplemental information) were non-significant, suggesting a broad accessibility for state anxiety reductions.

#### Flow and boredom as mediators of condition on anxiety reduction

**Figure 2:**
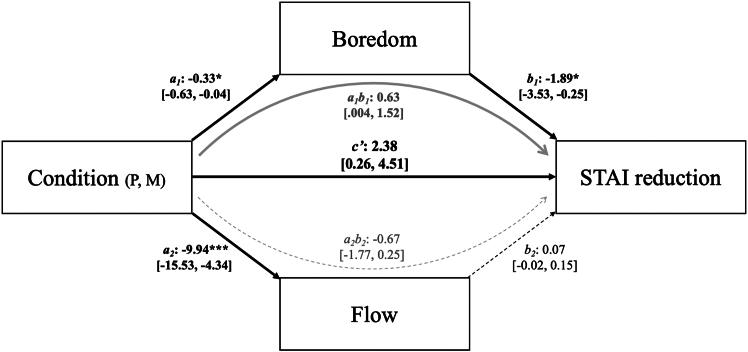
Repeated-measures parallel mediation of task condition on STAI reduction through boredom and flow P = Paint, M = Mazes. Boredom = Boredom (paint) – Boredom (mazes), Flow = Flow (paint) – Flow (mazes), STAI reduction = STAI reduction (paint) – STAI reduction (mazes); see Montoya and Hayes,^21^ p. 15. Bold reflects significance: ∗∗∗*p* < 0.001, ∗∗*p* < 0.01, and ∗*p* < 0.05 (note that the indirect effects do not have a *p* value due to the bootstrap/confidence-interval method of significance testing). Dashed reflects non-significance. Black reflects direct effects, light gray reflects indirect effects. See also Figures S1 and S2.

We next sought to evaluate whether flow and boredom could explain the relationship between Condition and anxiety reduction, and if so, how these variables differentially mediated this effect. To this end, we conducted a parallel (multiple) mediation model, including both Flow and Boredom as possible mediators of the effect of Condition (the repeated-measures independent variable) on STAI reduction scores (post- minus pre-condition STAI scores for each condition; the dependent variable) (Figure 2) (given the two-condition repeated-measures design, we used the MEMORE macro, version 2.1, available for SPSS^71^). This model thus jointly tests whether Flow, controlling for the effects of Boredom, uniquely explained anxiety reduction, and/or if Boredom, controlling for the effects of Flow, uniquely explained anxiety reduction. For sake of interpretation, we reversed the sign of STAI reduction scores in this particular analysis such that a positive score indicates a reduction in anxiety (i.e., affective improvement). Note that the computations involving the indirect effects do not have a *p* value due to the bootstrap method of significance testing; significance is instead determined by not containing 0 in the confidence intervals.

The total effect of Condition on STAI reduction score was significant (*c* = 2.34 [0.48, 4.21], *t*(95) = 2.49, *p* = 0.015), mirroring our original ANOVA interaction of Condition × Time. Condition significantly predicted Boredom such that the control condition was associated with higher boredom than painting (*a*_*1*_ = −0.33 [-0.63, −0.04], *t*(95) = −2.24, *p* = 0.027). Further, Boredom significantly negatively predicted STAI reduction (*b*_*1*_ = −1.89 [-3.53, −0.25], *t*(91) = −2.28, *p* = 0.025). Importantly, the indirect effect of Condition on STAI reduction score via Boredom (controlling for Flow), as generated through 10,000 bootstrap samples, was also significant (*a*_*1*_*b*_*1*_ = 0.63 [0.004, 1.52]).

Next, in the same model, Condition significantly predicted Flow such that, surprisingly, the control condition was associated with greater flow (*a*_*2*_ = −9.94 [-15.53, −4.34], *t*(95) = −3.53, *p* < 0.001). However, Flow did not significantly predict STAI reduction (*b*_*2*_ = 0.07 [˗0.02, 0.15], *t*(91) = 1.53, *p* = 0.13). Further, the indirect effect of Condition on STAI reduction via Flow (controlling for Boredom) was not significant (*a*_*2*_*b*_*2*_ = −0.67 [˗1.77, 0.25]). A contrast test comparing the indirect effect through Boredom and the indirect effect through Flow revealed that the indirect effect through Boredom was significantly larger than that through Flow (*a*_*1*_*b*_*1*_ – *a*_*2*_*b*_*2*_ = 1.30 [0.34, 2.14]). Lastly, the direct effect of Condition on STAI reduction, controlling for both Boredom and Flow, was significant (*c*’ = 2.38 [0.26, 4.51], *t*(91) = 2.23, *p* = 0.028).

In sum, the parallel mediation model revealed that Boredom (controlling for the effects of Flow) partially mediated the relationship between condition and STAI reduction (indirect pathway *a*_*1*_*b*_*1*_), such that higher levels of boredom were (1) experienced during the mazes condition relative to the painting condition (pathway *a*_*1*_) and (2) associated with greater levels of anxiety (pathway *b*_*1*_). In other words, individuals were more engaged during painting, and this engagement led to greater reductions in anxiety. Although, unexpectedly, mazes elicited greater flow experiences (pathway *a*_*2;*_ perhaps due to a more-optimal matching between challenge and skill in mazes than painting given the non-expert painting population), Flow did not significantly predict or mediate STAI reduction when controlling for the effects of Boredom (pathway *b*_*1*_; indirect pathway *a*_*2*_*b*_*2*_). Overall, we take these results as evidence of the power of active, creative engagement on emotion regulation.

### Physiological analyses

#### Condition differences

**Figure 3:**
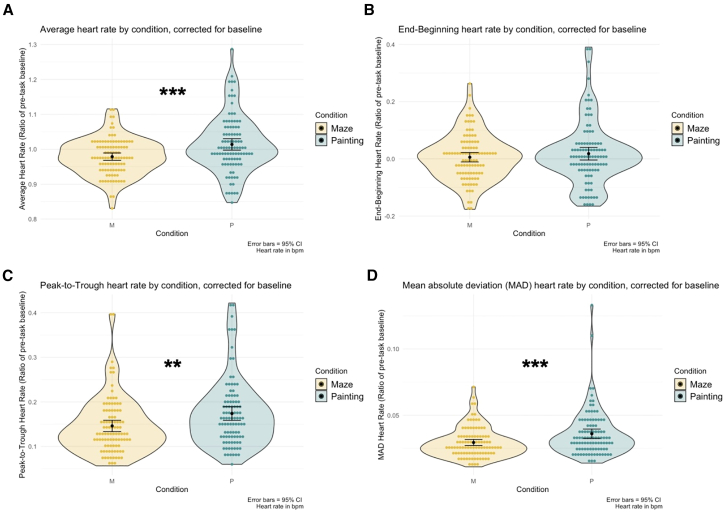
Differences in baseline-corrected heart rate measures between conditions reflect greater physiological reactivity during the creative painting task than the non-creative control task Significantly higher (A) Average, (C) Peak-to-Trough, and (D) MAD HR values emerged in painting than mazes. No significant difference was found in (B) End-beginning values. Data are represented as individual data points with superimposed mean ± 95% confidence intervals. ∗∗∗*p* < 0.001 and ∗∗*p* < 0.01. See also Figures S11 and S12.

We found significantly higher average HR in painting than mazes, *t*(97) = 3.67, *p* < 0.001, *d* = 0.38 (Figure 3A). End-beginning HR difference scores were not significantly different between conditions, *t*(97) = 0.90, *p* = 0.37 (Figure 3B). Peak-to-trough (maximum HR value minus minimum HR value) differences were significantly higher in painting than mazes, *t*(97) = 3.16, *p* = 0.002, *d* = 0.32 (Figure 3C). Lastly, mean absolute deviation (MAD)––which calculates overall volatility of HR across each 20-min task––was significantly higher in painting than mazes, *t*(97) = 3.59, *p* < 0.001, *d* = 0.36 (Figure 3D). A 2 (Condition: paint, maze) × 2 (Day: 1, 2) ANOVA intended to assess condition order on HR values for each of the four variables maintained significant condition effects (see supplemental information). Additionally, ANCOVAs controlling for the potential physiological confounds of recent exercise, caffeine, and substance use also maintained the significant condition effects (see supplemental information). These results suggest that painting elicited greater physiological reactivity than the active non-creative control condition, across multiple measures computed from continuous HR.

### Joint self-report and physiological analyses

Given the relationship between physiology and behavior in emotion processing, we examined how the self-report data interacted with the physiological results reported previously. These analyses included correlations between measures of HR and STAI scores and PCA-based moderation analyses (we did not examine HR as a mediating variable between Condition and anxiety reduction due to the theoretical uncertainty on the neurobiological roots of subjective emotion [i.e., if physiological signals engender, or simply are a manifestation of, specific emotions^72^^,^^73^]).

#### Physiology versus STAI difference scores

**Figure 4:**
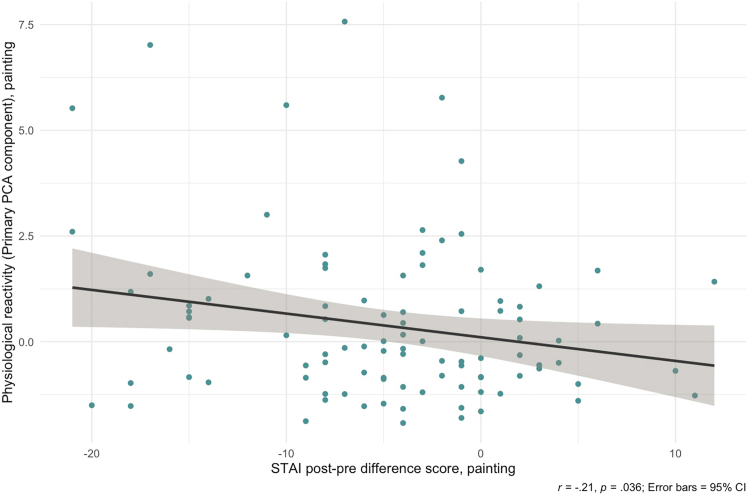
Greater reductions in self-reported anxiety associate with greater physiological reactivity during painting Scatterplot and a significant linear regression (*r* = −0.21, *p* = 0.036) between STAI difference scores from the painting condition and Physiological Reactivity––as measured by a principal components analysis (PCA) aggregating all pre-registered cardiac dependent variables––during the painting condition. Plot depicts individual data points +/− 95% confidence bands.

We first sought to correlate physiological activity during painting with the STAI difference scores. To do this, we used an aggregated measure of Physiological Reactivity derived from the four physiology dependent variables (see STAR Methods). A zero-order Pearson’s correlation test revealed a significant negative correlation between Physiological Reactivity during painting and STAI difference scores during painting, *r*(96) = −0.21, *p* = 0.036, suggesting that the greater physiological reactivity in the painting task, the greater the anxiety reduction (i.e., difference scores became more negative) (Figure 4). Thus, physiological reactivity significantly predicted the degree to which self-reported anxiety decreased during painting. We also note that, in response to a reviewer’s suggestion, we corrected for baseline anxiety levels by conducting a correlation between STAI difference scores (adjusted by dividing by pre-condition STAI scores) and Physiological Reactivity. A marginally significant correlation was observed, with a similar effect size and direction to those yielded by our initial analysis, *r*(96) = −0.19, *p* = 0.055. While this result supports the idea that physiological reactivity can predict reductions in self-reported anxiety reasonably well, the shift in *p* values before and after correcting for pre-condition STAI scores suggests that physiological reactivity’s predictive power of STAI reduction could be modestly influenced by baseline anxiety levels.

#### Trait-level moderation

We next examined whether certain trait-level variables significantly moderated the degree to which the painting elicited higher Physiological Reactivity. To accomplish this, we used aggregate measures of Psychopathology and General and Visual Creativity derived from trait-level questionnaires (see STAR Methods).

**Figure 5:**
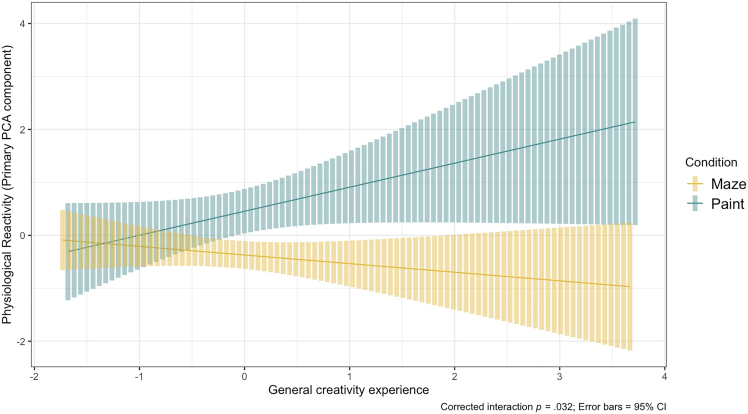
Greater general creativity experience associates with higher physiological reactivity during painting than during the non-creative control task As General Creativity increased, participants demonstrated greater Physiological Reactivity in painting compared to mazes, corrected *p* = 0.032, $\eta_{p}^{2}$ = 0.060. Plot depicts regressions based on individual data points +/− 95% confidence intervals. See also Figure S18.

Because two ANOVAs were conducted on the same dependent variable but with different moderators, we implemented Bonferroni corrections on the reported omnibus *p* values. Further details on the structure of these ANOVAs are found in the Method section. In the ANOVA with Psychopathology as a centered, between-subjects variable, the main effect of Condition was significant (as suggested by the physiology paired *t-*tests in Figure 3), *F*(1, 96) = 14.96, corrected *p* < 0.001, $\eta_{p}^{2}$ = 0.133. However, Psychopathology did not significantly moderate the main effect of Condition, *F*(1, 96) = 1.28, corrected *p* = 0.52, $\eta_{p}^{2}$ = 0.013. An ANOVA with General Creativity and Visual Creativity as centered, between-subjects variables revealed a significant main effect of Condition as before, *F*(1, 94) = 15.82, corrected *p* < 0.001, $\eta_{p}^{2}$ = 0.144. Interestingly, General Creativity significantly moderated the effect of Condition, *F*(1, 94) = 6.04, corrected *p* = 0.032, $\eta_{p}^{2}$ = 0.060. Post-hoc contrasts with Tukey corrections revealed that this effect was localized to a significant difference between conditions as General Creativity scores increased (at General Creativity = 0, i.e., the mean across participants, *t*(94) = −3.978, *p* < 0.001, *b* = −0.834; at General Creativity = 1, i.e., one standard deviation above the mean, *t*(94) = −4.223, *p* < 0.001, *b* = −1.451) (Figure 5). Visual Creativity did not moderate the effect of Condition, *F*(1, 94) = 0.12, corrected *p* = 1, $\eta_{p}^{2}$ = 0.001. The three-way interaction between General Creativity, Visual Creativity, and Condition was also non-significant, *F*(1, 94) = 0.91, corrected *p* = 0.684, $\eta_{p}^{2}$ = 0.010. These results suggest that experience in general creativity across domains (and not specific to the visual modality) is associated with greater physiological reactivity during a creative task like painting as compared to a non-creative task.

Lastly, we also explored whether trait-level mindfulness scores and openness scores moderated the degree to which painting elicited greater Physiological Reactivity. A similar moderation analysis was conducted, with both centered Mindfulness and centered Openness scores included as between-subjects variables. The analysis revealed a significant main effect of Condition (*F*(1, 94) = 13.37, *p* < 0.001, $\eta_{p}^{2}$ = 0.125), but a non-significant moderation of Mindfulness on Condition (*F*(1, 94) = 0.34, *p* = 0.561, $\eta_{p}^{2}$ = 0.004). However, a significant moderation effect of Openness on Condition did emerge *(F*(1, 94) = 6.00, *p* = 0.016, $\eta_{p}^{2}$ = 0.060), with the patterns of results mirroring the moderation effect of General Creativity: as Openness increased, Physiological Reactivity increased in painting relative to mazes (Figure S18). This suggests that not only does openness associate with greater physiological reactivity in creative tasks, but also that openness and general creativity experience map onto a similar trait relating to flexible thinking.

## Discussion

Art is valued as a unique expression of the human condition and is often accompanied by affective impacts on both the creator and consumer. However, due to the vague operationalization of “art” in study designs of art production research,^13^^,^^15^^,^^17^ open questions remain regarding the determinants and mechanisms by which art exerts a healing power more broadly. Here, we attempted to address these questions in a controlled, multi-modal study on the emotion regulation (ER) impact of abstract painting. To control for extraneous variables (e.g., task duration, experiment environment, motor movement) as well as intraindividual differences, each participant completed on separate days both the experimental abstract painting task and a non-creative, yet nonetheless active and engaging control task. We found that participants reported significant improvements across various mental health measures, but that painting selectively alleviated state anxiety relative to the maze-completion control condition (Figure 1A). This finding helps to pinpoint in what specific manner ER emerges during creative art production and adds to the growing body of work suggesting therapeutic benefits of engaging in art.

The significantly greater reduction in anxiety resulting from painting compared to the active control condition highlights a unique interaction between ER and creative activities like painting on selective aspects of mental health. Our results answer calls by researchers to examine effects of art production on anxiety,^74^ while also reinforcing other work (both in clinical and non-clinical samples) that has reported similar benefits on anxiety^27^^,^^62^^,^^75^ and stress^24^ from artistic activities. Importantly, extending Abbott et al.,^24^ we were able to attribute this effect more directly to the creative expression inherently involved in painting and not maze-completion. However, an interesting question remains as to why painting and mazes exhibited similar benefits for distress (SUDS) and for mood (POMS). Some researchers have argued that distress and anxiety are unique constructs, with distress being viewed as more somatic than anxiety, and anxiety being viewed as a more cognitively mediated state reflecting maladaptive future-oriented worry.^76^^,^^77^ Consistent with this view, studies have found participant reports of SUDS and STAI (while often positively correlated) do not capture equivalent variance (e.g., Bohensky et al.^78^). Alternatively, the differential impact of distress and anxiety may be attributable to method variance, given that SUDS is a simple one-item slider from 0 to 100 whereas STAI is a more comprehensive 20-item questionnaire (see Rossi & Pourtois^79^). It is also possible that, regardless of creative elements, any leisure activity (mazes or painting) equally benefits general distress or mood as indexed by the POMS, particularly when accompanied by a minimal degree of social interaction such as that provided by our experimental setting. For anxiety, however, the creative expression that is specific to art may minimize worries over external and future stressors, either by explicitly and creatively simulating and reframing their stressor via the art (see Addis et al.^52^), or simply by distracting them away from the stressor toward an engaging task.^28^ Future research would do well to continue exploring these questions.

We also assessed whether certain variables that were experienced during the tasks themselves helped explain the reduction in anxiety. In particular, we measured task engagement via flow and boredom. Flow occurs when an individual’s skill level is optimally matched to the challenge at hand, leading to a state of complete immersion and focused attention, which often drives feelings of reward.^32^^,^^34^^,^^36^^,^^37^^,^^38^^,^^39^ Conversely, boredom is thought to occur when skill surpasses task demand, creating an inverse relationship with flow.^41^^,^^42^^,^^44^ A parallel mediation model including both variables revealed that only Boredom significantly mediated the relationship between Condition and anxiety reduction, such that lower experienced boredom during painting yielded reduced anxiety. This finding corroborates claims of the maladaptive nature of boredom and its association with poor mental health.^47^^,^^80^^,^^81^^,^^82^ In particular, greater boredom is often characterized by meaninglessness and awareness of task-unrelated worries or self-worth^83^; this shift in attention may likely propagate feelings of anxiety, as suggested by our model.

Interestingly, in the presence of Boredom, there was no significant mediation by Flow. While a large literature suggests the beneficial affective power of flow in art production, which we could not corroborate, our results do mirror some work that has failed to report a predictive effect of flow on negative affect when placed in a model with other related variables like task enjoyment.^35^ One interesting finding from this model was that, while painting yielded greater anxiety reductions due to reduced rates of boredom than the control task, the control task nonetheless evoked greater flow states than painting. While initially surprising, this may be because flow is thought to emerge at an optimal level of skill and challenge: a level potentially learned through practice. Indeed, some of the Flow State Scale^84^ items probe this specific relationship between expertise and challenge (e.g., “My abilities matched the high challenge of the situation”). For non-expert painters, the novelty of the task may have disrupted this sense of alignment between their individual abilities and the demands of the task, resulting in lower flow scores in the painting condition. In contrast, the maze-completion task, which most individuals are more familiar with, may have better matched participants’ perceived skill levels, thereby eliciting higher self-reported flow. Thus, painting may have been less boring due to the task challenge, but perhaps too challenging for this sample such that it hindered the ability to achieve a flow state. In sum, our model reveals that boredom and flow are more distinct than previously thought^29^ (see Ulrich et al.^85^), with boredom emerging as a potential hinderance in behavioral approaches to ER.

Notably, our study also revealed that measures of physiological reactivity correspond with the self-reported anxiety reductions. Across four pre-registered variables computed from baseline-corrected HR, three revealed greater physiological reactivity during painting than in the control task (Figure 3), and do not appear to be attributed to task differences in motor movement. Further, a general Physiological Reactivity component aggregated across these individual measures of HR (thus capturing diverse profiles of cardiac functioning) significantly correlated with the magnitude of anxiety reductions during painting (Figure 4) (although a supplemental analysis revealed that anxiety scores at baseline may influence the significance of this association). This result converges with the art-making study of Sandmire et al.,^27^ who found that as physiological signaling (in their case, heart rate reactivity) increased, anxiety reduced. Interestingly, the Physiological Reactivity in our paper seemed to be systematically trained with greater experiences in creativity-related domains (Figure 5), as well as trait-level openness to experience. In other words, self-reported creative experience and openness to experience yielded similar predictive effects of physiological reactivity, replicating and extending claims that openness is a reliable marker for creativity.^20^

One explanation for this expertise-related finding could be that these creative individuals demonstrate increased interest or effort in creative tasks, and thus physiologically respond in a more excited manner.^86^ Alternatively, perhaps these creative individuals have naturally learned the adequate levels of engagement needed in a creative task to extrapolate emotional benefits (though, we note that neither creativity experience nor trait-level psychopathology moderated the degree of self-reported anxiety reduction itself, see Data S7). Nonetheless, the finding that physiological reactivity increased during an emotionally therapeutic experience warrants further discussion, particularly in relation to the “meditation paradox.”^67^ Although elevated HR is often associated with anxiety and sympathetic nervous system arousal, as mentioned earlier, there are well-documented cases in which increased physiological activity co-occurs with emotional downregulation, such as during physical exercise and cognitively effortful forms of meditation.^68^^,^^69^^,^^70^ Our findings suggest that art-making may function similarly: rather than promoting relaxation through passivity or stillness, art-making may support ER through active cognitive and physiological engagement. More broadly, this aligns with the idea that therapeutic benefit can emerge from heightened, rather than dampened, neural and physiological activation, as seen in mindfulness-based therapies^87^ and interventions such as high-frequency transcranial magnetic stimulation.^88^

### Limitations of the study

While our results offer an optimistic avenue for future interventions, there are some notable issues that are worthy of consideration. First, we did not specifically target individuals diagnosed with affective disorders, which could raise concerns about the applicability of our findings to clinical populations. That is, our experiment was not specifically a clinical art-therapy intervention. However, despite not targeting a clinical sample, many of our participants exhibited anxiety levels above the clinical cutoff^89^ (see Figure S3). Additionally, trait-level psychopathology did not moderate the anxiety reduction from painting, indicating that the benefits of painting are similarly strong across varying degrees of psychopathology, which in turn suggests that our pattern of results might well obtain in a clinical sample. In any case, our findings provide meaningful information for managing anxiety or stress in the general population, which is beneficial as it demonstrates applicability to a large number of people, even those without clinical conditions. Furthermore, these findings highlight the potential for simple and cost-effective mental health initiatives.

Another consideration of our study is the use of Fitbit devices to track physiological reactivity. While Fitbits have been validated for HR measurements,^90^^,^^91^^,^^92^ future work would benefit from measuring HR variability as well. Values of momentary HR variability (and its corresponding interbeat interval timeseries data) are not released from Fitbits, but may provide more nuanced insights into cardiovascular function and a more sensitive measure of emotional resilience (e.g., Neacsiu et al.^93^; see Appelhans and Luecken^94^). Nonetheless, the portability and non-invasive nature of Fitbits provided a benefit that other gold-standard devices may not have been able to offer without altering the naturalistic artistic process.

A final point worth considering is the reliance on our maze-completion control task to support our claims of anxiety reduction. Critics might argue that solving mazes involves some creative thinking, as it requires participants to visualize various possible pathways to the solution. However, we contend that the creativity in abstract painting involves a more authentic form of creative thinking, characterized by generating multiple unique and original ideas, which is not present in maze-solving. Nevertheless, future research should include additional active control conditions, such as those manipulating social interaction levels—given the emphasis on social bonding in many art therapy sessions^15^—or exploring other forms of creative engagement, like simple art observation.

### Conclusion

In sum, our study addresses many of the calls for interpretable research on the emotional benefits of art production by incorporating within-person variability, multi-modal data to determine holistic determinants and correlates of creative art production, and multiple conditions to control for extraneous variables. Specifically, we found a selective effect of anxiety reduction from painting compared to an active but non-creative leisure activity, while also observing overall reductions in distress and improvements in mood. Self-reported boredom and objective physiological data provided further insights into the anti-anxiety benefits of painting. Thus, while the aesthetics literature continues to explore the unique processes underlying creative activities, our study highlights a promising future direction at the intersection of emotion regulation, anxiety, and art production.

## Resource availability

### Lead contact

Further information and requests for resources should be directed to and will be fulfilled by the lead contact, Kevin LaBar (klabar@duke.edu).

### Materials availability

This study did not generate new unique reagents.

### Data and code availability

• Behavioral and physiological data that support the findings of this paper have been deposited at https://osf.io/4cqzx/. They are publicly available as of the date of publication. The DOI is listed in the key resources table.
• All original code has been deposited at https://osf.io/4cqzx/ and is publicly available as of the date of publication. The DOI is listed in the key resources table.
• Any additional information required to reanalyze the data reported in this paper is available from the lead contact upon request.

## Acknowledgments

The authors thank Leonard Faul for his assistance in statistical analyses, Grant Shields for his expertise in stress-related study designs, Gregory Samanez-Larkin for providing the Fitbit equipment, and Tom Szigethy for his expertise in student-health interventions. This work was supported by a 10.13039/100006510Duke University Bass Connections Student Research Award (L.B., C.W., and P.S.). L.B. was supported by the 10.13039/100023581National Science Foundation Graduate Research Fellowship Program.

## Author contributions

Conceptualization and methodology, L.B., K.S.L., and P.S.; funding acquisition, L.B., C.W., and P.S.; investigation, L.B., K.L., C.W., and J.C.; formal analysis, L.B. and K.L.; writing – original draft, L.B. and K.L.; writing – review and editing, L.B., K.S.L., and P.S.; supervision: K.S.L. and P.S.

## Declaration of interests

The authors declare no competing interests.

## STAR★Methods

### Key resources table

**Table:** 

REAGENT or RESOURCE	SOURCE	IDENTIFIER
**Deposited data**		

Self-report questionnaire data of experiment	This article	https://doi.org/10.17605/OSF.IO/4CQZX
Physiological data of experiment	This article	https://doi.org/10.17605/OSF.IO/4CQZX

**Software and algorithms**		

R	R Project	https://www.r-project.org/
SPSS	IBM SPSS Statistics	https://www.ibm.com/products/spss-statistics

**Other**		

Preregistration	This article	https://doi.org/10.17605/OSF.IO/4CQZX

### Experimental model and study participant details

One-hundred participants were recruited via the Duke University undergraduate participant pool (for course credit) and through an online bulletin (for $40 payment) open to all campus affiliates, including undergraduate and graduate students, and staff. Individuals taking cardiovascular medication were excluded from data recruitment to minimize physiological confounds^95^; student athletes were also ineligible to enroll for similar reasoning.^96^ Participants needed to own a smartphone able to download the Fitbit mobile application, though we note that no individual was denied participation because they did not own a smartphone. Power analyses were not performed *a priori*; instead, we reviewed the most closely related behavioral or psychophysiological affective-related aesthetics studies and found that 72 adults were recruited in Bozcuk et al.,^9^ 50 adults in Haiblum-Itskovitch et al.,^97^ and 70 in Forkush and Drake.^34^ To ensure higher power, we increased upon these sample sizes and also implemented a repeated-measures cross-over design. One participant was excluded from all data analyses for failing to follow instructions (i.e., painting a representational image), leading to a final sample size of 99 (*M* = 21.18, *SD* = 4.22; female = 72). Sixty-five of these individuals were recruited through the undergraduate participant pool, while 34 were recruited through the on-line campus bulletin. Participants were of the following racial/ethnic backgrounds: 26 White, 19 South Asian, 17 East or Southeast Asian, 11 Black or African-American, 10 Latine, 4 Middle Eastern, 1 Native Hawaiian or Pacific Islander, 7 bi- or multi-racial, 1 other, and 3 who preferred not to indicate. Due to technological difficulties with one Fitbit device leading to spurious and invalid data, the physiological data from an additional participant was excluded but their behavioral data were maintained in analyses.

### Method details

Participants were told at sign-up that the study was to take place across three consecutive days, with the first two days in-person and the third day’s tasks administered remotely. There was no mention of artistic production or creativity during recruitment. They were informed that the study examined how different leisure activities affected subjective reports of mood and physiological responses as measured through a heart-rate tracker. Once the participants provided written, informed consent, the experimenter fit the participant with the lab-provided Fitbit Charge 4 device on his/her non-dominant wrist, and subsequently set up the connection between the device and the participant’s cell phone on which the Fitbit application was downloaded. Participants logged into the application via a lab account. Lastly, the experimenter connected the participant’s lab account to the Fitabase aggregation software,^98^ which exported the continuous physiological data from the Fitbit.

Following device set-up, participants began a Qualtrics survey on the lab computer. First, participants completed ten trait-level questionnaires, followed by three state-level mood questionnaires and additional questions about task experience and possible physiological moderators within the last 24 hours (e.g., exercise, caffeine consumption, substance use). Upon completion of these questionnaires, participants were randomly assigned by the Qualtrics program to the abstract painting task or the control task, which was a series of printed mazes meant to be completed with a pencil. We selected this control task to match motor movement of the wrist as much as possible across conditions (a paired *t*-test done on calories exerted [as measured by the Fitbit device] found no significant differences between calories burned on the day of painting and on the day of the maze task, *t*(97) = -0.74, *p* = 0.46), while invoking little to no creative expression. The experimenter informed the participants that they would have 20 minutes for the given task and provided them the proper materials.

For the abstract painting task, we followed a similar procedure from Bellaiche, Smith, et al.^66^ which originally validated the use of abstract painting as a proxy for creative ability. Participants were given 13 ink colors, an apron, five brushes, three palette knives, a water cup, and an 11 x 14″ blank canvas. As in Bellaiche, Smith, et al.,^66^ the experimenter defined an abstract painting as “a painting that does not represent images of our everyday world. It has lines and shapes, but they are not meant to represent objects or living things.” Lastly, mirroring clinical implementations of art therapy,^99^ participants were encouraged to express their thoughts and emotions as they wished. The experimenter reiterated that that no other individuals would see their work except the experimenters, and that “this is your own space to engage in this leisure activity.” Printed instructions were also left in the room during the task.

For the control task, participants were given a small stack of printed mazes meant to be completed serially. The experimenter emphasized that we would not assess the accuracy, speed, or number of mazes completed in the 20 minutes, and to accordingly complete the task without pressure. We reiterated that no other individuals would see their work––including experimenters, who discarded the completed mazes–– and that “this is your own space to engage in this leisure activity.” Printed instructions were again left in the room during the task.

At the conclusion of the 20 minutes, the experimenter instructed the participants to complete a series of post-condition questionnaires, which included the same state-level mood questionnaires, as well as measures of flow state, boredom, perception of task performance, and likelihood they would engage in a future creative task. Finally, the participant returned his/her Fitbit to the experimenter, who reminded them to return the next day.

On the second day, participants reconnected their cell phones to the same Fitbit device as before, and they completed the state-level mood questionnaires and questions asking about recent physiological confounds before being assigned the condition that they had not completed the previous day. The condition was administered with the respective procedure described above. At the conclusion of the 20-minute task, participants completed the state-level questionnaires once again. When finished, the experimenter disconnected the Fitbit device from the participants’ cell phone and logged them out of the mobile Fitbit application. Before leaving, the participant reminded them to complete the online questionnaire the next day.

On the third and final day of the study, participants completed an online survey with the state-level questionnaires once again. Upon completion of this survey, they were debriefed on the nature of the study and were provided with details on their compensation.

#### Materials

##### Self-report

On the first day of the study, trait-level questionnaires were administered to measure a range of traits related to emotion processing, creativity, and/or psychopathology. See Method S1 for a full list of all questionnaires. We analyzed results from the Inventory of Creative Activities and Achievements^20^ as a measure of creative experience (modality-specific and aggregated across modalities) (Figure S4), as well as the trait-level portion of the State-Trait Anxiety Inventory^100^ (STAI; Figure S3A) questionnaire and the 21-item Depression, Anxiety, and Stress Scale^101^ (Figures S3B and S3C) as measures of trait-level mental health. In response to reviewer suggestions, we also explored scores from the Mindfulness Attention Awareness Scale (MAAS) as a measure of trait mindfulness,^102^ as well as openness to experience scores from the NEO Five-Factor Inventory.^103^

As our primary dependent variables, three questionnaires indexing state-level affect were administered immediately before and after each condition, as well as on the third day remotely. To capture state-level anxiety, we administered the state portion of the STAI,^100^ summed and reverse-scored where appropriate (Figure S5). To measure total mood disturbance, participants completed the 40-item Profile of Mood States^104^ (POMS). In accordance with the scoring instructions, total mood disturbance was calculated as the difference between the summed negative subscales (Tension, Depression, Anger, Fatigue, Confusion) and the summed positive subscales (Vigor, Esteem-related Affect), with items reverse-scored where appropriate. Lastly, to measure general distress, participants completed the Subjective Units of Distress Scale, which is a one-item slider ranging from 0 (totally relaxed) to 100 (Highest distress ever felt).^105^

In addition, we wished to assess potential mechanisms driving changes in affect from each condition. In particular, we sought to capture and compare between experiences of flow state and of boredom, as the exact relationship between these variables is unclear (e.g., flow reports sometimes but do not always discriminate from boredom reports^85^), but both are nonetheless thought to be meaningful variables underlying task engagement.^29^^,^^41^^,^^42^^,^^44^ Methodologically, because these state-level variables are theoretically thought to *engender* emotion-related outcomes,^37^ they are well suited to be tested via mediation models (e.g., Rogatko^39^; Tse et al^106^) above and beyond correlational regression models (e.g., Genuth & Drake^35^). To capture Total Flow experienced during each condition, we administered the 36-item Flow State Scale^84^ (see Method S1, Table S1). To measure Boredom, we administered a single-item boredom question (“Using the following scale, please indicate how boring you thought the leisure activity you were asked to do was”) on a 1-5 Likert scale. Due to participant error, flow state and boredom data from three participants were not collected, leading to a sample of 96 for mediation analyses only.

Lastly, participants answered several other exploratory, single-item questions after each condition (e.g., Figure S17), including likelihood to engage in future activities and perception of task performance.

##### Physiological tracking

To measure the physiological effects from the conditions, we selected the HR monitor Fitbit Charge 4 for its portability, ease of use, and validated data (see Method S2). Our cross-over design was designed to measure relative *changes* in HR across conditions within-subjects rather than raw, absolute HR values themselves. Nonetheless, we also corrected for individual baseline HR values in our physiological analyses (see Method S3; Figures S11 and S12).

The Fitbit device samples average HR (in bpm) every 5-15 seconds, and then calculates an average HR each minute based on these values. Our calculations utilize this minute-by-minute sampling rate. Our study specifically pre-registered and analyzed four different measures of HR to capture as diverse of HR reactivity profiles as possible during the 20-minute conditions. Average HR referred to the average value across the condition duration, and was thus a metric of central tendency. End-Beginning (difference in HR between the end and start of the condition) and Peak-to-Trough (difference in maximum and minimum HR value across the condition duration) involved subtracting levels of HR at two timepoints, thus capturing more-detailed aspects of reactivity. Lastly, Mean Absolute Deviation (MAD) assessed volatility of HR by summing the differences between HR at minute *t* and Average HR across the condition, at each minute *t*. To statistically assess the differences in physiological reactivity between conditions, we conducted paired *t*-tests on each of these baseline-corrected HR measures. Physiological data from one participant were removed from analyses due to technological difficulties with the Fitbit device, leading to a sample of 98 participants only for physiological analyses.

### Quantification and statistical analysis

Our analysis procedure followed nearly identically from that listed in our pre-registration; significant deviations are noted. All statistical tests except for the mediation analyses were done in R, version 4.3.2. Tests of analysis of variance (ANOVA) were carried out with the *afex* package, version 1.3.0. Post hoc contrasts were carried out using the *emmeans* package, version 1.9.0. To maintain conservative results, all post-hoc contrasts were two-tailed and implemented Tukey corrections with pairwise method unless otherwise stated. Mediation analyses were done in SPSS, version 29.0.0.0. Statistical significance is denoted by *p*-values below 0.05.

#### Principle component analyses

##### Trait-level moderations

To assess how various self-reported, trait-level questionnaire data may moderate our main findings (both self-reported STAI and physiology results), we conducted a targeted principal components analysis (PCA) to derive three trait factors that may influence individual emotion- and/or creativity-related processing: Psychopathology, General Creativity background, and Visual Creativity background. This specific PCA procedure was not listed in our pre-registration but served to assuage concerns of inflated *p*-values with multiple comparisons. All PCAs were conducted using the *prcomp* function with scaling and centering from the default R *stats* package, version 4.3.2. No additional rotation methods were implemented given that we only maintained the first component from each PCA. Scree plot and specific loadings of variables are specified in the supplemental information. One PCA was performed to extract an underlying Psychopathology factor based on scores across questionnaires. Specifically, we fed the model each of the Depression, Anxiety, and Stress subscores from the DASS-21^101^ in addition to scores of the STAI (Trait) questionnaire.^100^ One principal component explaining 71.7% of the variance was obtained (Figures S8 and S13). A second PCA was performed to extract general creative behavior based on scores from the ICAA questionnaire.^20^ Specifically, we fed the model the General Creativity: Achievements and General Creativity: Activities items from the ICAA. One principal component explaining 83% of the variance was obtained (Figures S10 and S15). A third and final PCA was performed to extract creative behavior specific to the visual domain based on scores from the ICAA questionnaire.^20^ Specifically, we fed the model the Visual Achievements, Visual Activities, and Years of Visual Activities subscores from the ICAA. One principal component explaining 69.2% of the variance was obtained (Figures S9 and S14).

Each of the above PCAs produced a single score for each individual, which we then centered and added to repeated-measures ANOVAs as between-subjects variables, with Condition (Paint, Maze) as the within-subject variable and state STAI scores as the dependent variable. Specifically, we performed two such ANOVAs: one with Psychopathology included, and one with both General Creativity and Visual Creativity included. This latter ANOVA was designed as such given that the variables loaded into the General Creativity PCA component (General Achievements, General Activities) were originally aggregated scores across creativity domains from the ICAA questionnaire, which included visual modality questions also loaded into the Visual Creativity PCA component. Thus, an ANOVA with both factors considers the unique variance offered by visual and non-visual creativity.

##### Physiological reactivity

To minimize the number of tests conducted, we also conducted a PCA to aggregate across the four physiology dependent variables (Average, Peak-to-Trough, End-Beginning, and MAD) into one general Physiological Reactivity variable. This PCA method was not specified in our pre-registration but again served to reduce the number of statistical tests conducted via data reduction of this complex dataset. The primary retained component of this Physiological Reactivity PCA explained 64.1% of the variance (Figure S16).

This aggregated Physiological Reactivity variable was added as the dependent variable of two repeated-measures ANOVAs with Condition (Paint, Maze) as the within-subjects variable. Specifically, in these ANOVAs, we included the earlier trait-level PCA factors (Psychopathology, General Creativity, Visual Creativity) to test for a potential moderation effect of these variables on the significant relationship between Condition and Physiological Reactivity As before, we performed two such ANOVAs with Physiological Reactivity as the dependent variable: one with Psychopathology included, and one with both General Creativity and Visual Creativity included.

### Additional resources

The study design and analysis plan were pre-registered; the pre-registration can be found at https://osf.io/4cqzx/. The study protocol was approved by the Duke University Campus Institutional Review Board (2022-0463).

## References

[1] Graf L.K.M., Landwehr J.R. (2015). A dual-process perspective on fluency-based aesthetics: The pleasure-interest model of aesthetic liking. Pers. Soc. Psychol. Rev..

[2] Knapp R.H., Wulff A. (1963). Preferences for Abstract and Representational Art. J. Soc. Psychol..

[3] Keltner D., Haidt J. (2003). Approaching awe, a moral, spiritual, and aesthetic emotion. Cogn. Emot..

[4] Pelowski M., Markey P.S., Forster M., Gerger G., Leder H. (2017). Move me, astonish me, delight my eyes and brain: The Vienna Integrated Model of top-down and bottom-up processes in Art Perception (VIMAP) and corresponding affective, evaluative, and neurophysiological correlates. Phys. Life Rev..

[5] Chatterjee A., Vartanian O. (2016). Neuroscience of aesthetics. Ann. N. Y. Acad. Sci..

[6] Leder H., Belke B., Oeberst A., Augustin D. (2004). A model of aesthetic appreciation and aesthetic judgments. Br. J. Psychol..

[7] Sherman A., Morrissey C. (2017). What Is Art Good For? The Socio-Epistemic Value of Art. Front. Hum. Neurosci..

[8] Kant I. (2001).

[9] Bozcuk H., Ozcan K., Erdogan C., Mutlu H., Demir M., Coskun S. (2017). A comparative study of art therapy in cancer patients receiving chemotherapy and improvement in quality of life by watercolor painting. Complement. Ther. Med..

[10] Fancourt D., Garnett C., Spiro N., West R., Müllensiefen D. (2019). How do artistic creative activities regulate our emotions? Validation of the Emotion Regulation Strategies for Artistic Creative Activities Scale (ERS-ACA). PLoS One.

[11] Fancourt D., Finn S. (2019). http://www.ncbi.nlm.nih.gov/books/NBK553773/.

[12] Gruber H., Oepen R. (2018). Emotion regulation strategies and effects in art-making: A narrative synthesis. Arts Psychother..

[13] Martin L., Oepen R., Bauer K., Nottensteiner A., Mergheim K., Gruber H., Koch S.C. (2018). Creative Arts Interventions for Stress Management and Prevention—A Systematic Review. Behav. Sci..

[14] Sharp K., Hewitt J. (2014). Dance as an intervention for people with Parkinson’s disease: A systematic review and meta-analysis. Neurosci. Biobehav. Rev..

[15] Uttley L., Scope A., Stevenson M., Rawdin A., Taylor Buck E., Sutton A., Stevens J., Kaltenthaler E., Dent-Brown K., Wood C. (2015). Art therapy for people with non-psychotic mental disorders. Health Technol. Assess..

[16] Van Lith T., Schofield M.J., Fenner P. (2013). Identifying the evidence-base for art-based practices and their potential benefit for mental health recovery: A critical review. Disabil. Rehabil..

[17] Skov M., Nadal M. (2025). Can arts-based interventions improve health? A conceptual and methodological critique. Phys. Life Rev..

[18] Glück J., Ernst R., Unger F. (2002). How Creatives Define Creativity: Definitions Reflect Different Types of Creativity. Creat. Res. J..

[19] Alperson P., Levinson J. (2005). The Oxford Handbook of Aesthetics.

[20] Diedrich J., Jauk E., Silvia P.J., Gredlein J.M., Neubauer A.C., Benedek M. (2018). Assessment of real-life creativity: The Inventory of Creative Activities and Achievements (ICAA). Psychology of Aesthetics, Creativity, and the Arts.

[21] Ivcevic Z. (2009). Creativity map: Toward the next generation of theories of creativity. Psychology of Aesthetics, Creativity, and the Arts.

[22] Le Rhun A., Caillet P., Lebeaupin M., Duval M., Guilmault L., Anthoine E., Borghi G., Leclère B., Moret L. (2023). Mind-body and art therapies impact on emotional regulation in patients with chronic diseases: A pragmatic mixed-methods randomized controlled trial. BMC Complement. Med. Ther..

[23] Case C., Dalley T. (2014).

[24] Abbott K.A., Shanahan M.J., Neufeld R.W.J. (2013). Artistic Tasks Outperform Nonartistic Tasks for Stress Reduction. Art Ther. (Alex)..

[25] Kaimal G., Ray K., Muniz J. (2016). Reduction of Cortisol Levels and Participants’ Responses Following Art Making. Art Ther..

[26] Sandmire D.A., Gorham S.R., Rankin N.E., Grimm D.R. (2012). The Influence of Art Making on Anxiety: A Pilot Study. Art Ther. (Alex)..

[27] Sandmire D.A., Rankin N.E., Gorham S.R., Eggleston D.T., French C.A., Lodge E.E., Kuns G.C., Grimm D.R. (2016). Psychological and autonomic effects of art making in college-aged students. Anxiety Stress Coping.

[28] Fancourt D., Ali H. (2019). Differential use of emotion regulation strategies when engaging in artistic creative activities amongst those with and without depression. Sci. Rep..

[29] Csikszentmihalyi M. (1975).

[30] Dietrich A. (2004). Neurocognitive mechanisms underlying the experience of flow. Conscious. Cogn..

[31] de Manzano Ö., Theorell T., Harmat L., Ullén F. (2010). The psychophysiology of flow during piano playing. Emotion.

[32] Nakamura J., Csikszentmihalyi M., Snyder C.R., Lopez S.J. (2002). Handbook of Positive Psychology.

[33] Tan J., Di Bernardi Luft C., Bhattacharya J. (2023). The After-Glow of Flow: Neural Correlates of Flow in Musicians. Creat. Res. J..

[34] Forkosh J., Drake J.E. (2017). Coloring Versus Drawing: Effects of Cognitive Demand on Mood Repair, Flow, and Enjoyment. Art Therapy.

[35] Genuth A., Drake J.E. (2021). The benefits of drawing to regulate sadness and anger: Distraction versus expression. Psychology of Aesthetics, Creativity, and the Arts.

[36] Collier A.F., von Károlyi C. (2014). Rejuvenation in the “making”: Lingering mood repair in textile handcrafters. Psychology of Aesthetics, Creativity, and the Arts.

[37] Kapitan L. (2013). Art Therapy’s Sweet Spot Between Art, Anxiety, and the Flow Experience. Art Therapy.

[38] Rankin K., Walsh L.C., Sweeny K. (2019). A better distraction: Exploring the benefits of flow during uncertain waiting periods. Emotion.

[39] Rogatko T.P. (2009). The Influence of Flow on Positive Affect in College Students. J. Happiness Stud..

[40] Warren S. (2006). An exploration of the relevance of the concept of “flow” in art therapy. Int. J. Art Ther..

[41] Raffaelli Q., Mills C., Christoff K. (2018). The knowns and unknowns of boredom: A review of the literature. Exp. Brain Res..

[42] Weibel D., Wissmath B., Bieleke M., Wolff W., Martarelli C. (2024). The Routledge International Handbook of Boredom.

[43] Dewaele J.-M., Macintyre P., Kamal Ahmed I., Albakistani A. (2025). Emotional, Attitudinal, and Sociobiographical Sources of Flow in Online and In-Person EFL Classrooms. Appl. Linguist..

[44] Harris M.B. (2000). Correlates and Characteristics of Boredom Proneness and Boredom. J. Appl. Soc. Psychol..

[45] Weybright E.H., Doering E.L., Perone S. (2022). Difficulties with Emotion Regulation during COVID-19 and Associations with Boredom in College Students. Behav. Sci..

[46] Crockett A.C., Myhre S.K., Rokke P.D. (2015). Boredom proneness and emotion regulation predict emotional eating. J. Health Psychol..

[47] Milea I., Cardoş R.A.I., David D. (2021). The map of cognitive processes in boredom: Multiple mediation models. Behav. Cogn. Psychother..

[48] Lepera N. (2011). Relationships between boredom proneness, mindfulness, anxiety, depression, and substance use. New Sch Psychol Bull.

[49] Nederkoorn C., Vancleef L., Wilkenhöner A., Claes L., Havermans R.C. (2016). Self-inflicted pain out of boredom. Psychiatry Res..

[50] Forzoni S., Perez M., Martignetti A., Crispino S. (2010). Art therapy with cancer patients during chemotherapy sessions: An analysis of the patients’ perception of helpfulness. Palliat. Support Care.

[51] Blomdahl C., Gunnarsson A.B., Guregård S., Björklund A. (2013). A realist review of art therapy for clients with depression. Arts Psychother..

[52] Addis D.R., Pan L., Musicaro R., Schacter D.L. (2016). Divergent thinking and constructing episodic simulations. Memory.

[53] Kaimal G. (2019). Adaptive Response Theory: An Evolutionary Framework for Clinical Research in Art Therapy. Art Therapy.

[54] Volpe D., Signorini M., Marchetto A., Lynch T., Morris M.E. (2013). A comparison of Irish set dancing and exercises for people with Parkinson’s disease: A phase II feasibility study. BMC Geriatr..

[55] Schwarz N. (1999). Self-reports: How the questions shape the answers. Am. Psychol..

[56] Shields G.S. (2020). Stress and cognition: A user’s guide to designing and interpreting studies. Psychoneuroendocrinology.

[57] Turner J.R. (1994).

[58] Deutz M.H.F., Woltering S., Vossen H.G.M., Deković M., van Baar A.L., Prinzie P. (2019). Underlying Psychophysiology of Dysregulation: Resting Heart Rate and Heart Rate Reactivity in Relation to Childhood Dysregulation. J. Am. Acad. Child Adolesc. Psychiatry.

[59] Grogan E.L., Norris P.R., Speroff T., Ozdas A., France D.J., Harris P.A., Jenkins J.M., Stiles R., Dittus R.S., Morris J.A. (2005). Volatility: A New Vital Sign Identified Using a Novel Bedside Monitoring Strategy. J. Trauma.

[60] Grogan E.L., Morris J.A., Norris P.R., France D.J., Ozdas A., Stiles R.A., Harris P.A., Dawant B.M., Speroff T. (2004). Reduced Heart Rate Volatility: An Early Predictor of Death in Trauma Patients. Ann. Surg..

[61] Regehr C., Glancy D., Pitts A. (2013). Interventions to reduce stress in university students: A review and meta-analysis. J. Affect. Disord..

[62] Ashlock L.E., Miller-Perrin C., Krumrei-Mancuso E. (2019). The effectiveness of structured coloring activities for anxiety reduction art therapy. Art Therapy.

[63] Mohammadian Y., Shahidi S., Mahaki B., Mohammadi A.Z., Baghban A.A., Zayeri F. (2011). Evaluating the use of poetry to reduce signs of depression, anxiety and stress in Iranian female students. Arts Psychother..

[64] Bittman B.B., Snyder C., Bruhn K.T., Liebfreid F., Stevens C.K., Westengard J., Umbach P.O. (2004). Recreational Music-making: An Integrative Group Intervention for Reducing Burnout and Improving Mood States in First Year Associate Degree Nursing Students: Insights and Economic Impact. Int. J. Nurs. Educ. Scholarsh..

[65] Isis P., Bokoch R., Fowler G., Hass-Cohen N. (2023). Efficacy of a Single Session Mindfulness-based Art Therapy Doodle Intervention. Art Therapy.

[66] Bellaiche L., Smith A.P., Barr N., Christensen A., Williams C., Ragnhildstveit A., Schooler J., Beaty R., Chatterjee A., Seli P. (2023). Back to the basics: Abstract painting as an index of creativity. Creat. Res. J..

[67] Peng C.-K., Henry I.C., Mietus J.E., Hausdorff J.M., Khalsa G., Benson H., Goldberger A.L. (2004). Heart rate dynamics during three forms of meditation. Int. J. Cardiol..

[68] Lumma A.-L., Kok B.E., Singer T. (2015). Is meditation always relaxing? Investigating heart rate, heart rate variability, experienced effort and likeability during training of three types of meditation. Int. J. Psychophysiol..

[69] Carter T., Morres I., Repper J., Callaghan P. (2016). Exercise for adolescents with depression: Valued aspects and perceived change. J. Psychiatr. Ment. Health Nurs..

[70] Lam L.C.W., Riba M. (2016).

[71] Montoya A.K., Hayes A.F. (2017). Two-condition within-participant statistical mediation analysis: A path-analytic framework. Psychol. Methods.

[72] Gendron M., Barrett L.F. (2009). Reconstructing the Past: A Century of Ideas About Emotion in Psychology. Emot. Rev..

[73] James W. (1922).

[74] Abbing A., Ponstein A., van Hooren S., de Sonneville L., Swaab H., Baars E. (2018). The effectiveness of art therapy for anxiety in adults: A systematic review of randomised and non-randomised controlled trials. PLoS One.

[75] van der Vennet R., Serice S. (2012). Can Coloring Mandalas Reduce Anxiety? A Replication Study. Art Therapy.

[76] Berde C., Wolfe J. (2003). Pain, anxiety, distress, and suffering: Interrelated, but not interchangeable. J. Pediatr..

[77] Tuma A.H., Maser J.D. (1985).

[78] Bohensky M., Johnson A.T., Vossoughi J. (2017). Effect of Induced Anxiety on Respiratory Resistance Using Virtual Reality Simulation. Open J. Respir. Dis..

[79] Rossi V., Pourtois G. (2012). Transient state-dependent fluctuations in anxiety measured using STAI, POMS, PANAS or VAS: A comparative review. Anxiety Stress Coping.

[80] Goldberg Y.K., Eastwood J.D., LaGuardia J., Danckert J. (2011). Boredom: An Emotional Experience Distinct from Apathy, Anhedonia, or Depression. J. Soc. Clin. Psychol..

[81] Nett U.E., Goetz T., Hall N.C. (2011). Coping with boredom in school: An experience sampling perspective. Contemp. Educ. Psychol..

[82] Weiss E.R., Todman M., Maple E., Bunn R.R. (2022). Boredom in a Time of Uncertainty: State and Trait Boredom’s Associations with Psychological Health during COVID-19. Behav. Sci..

[83] Eastwood J.D., Frischen A., Fenske M.J., Smilek D. (2012). The Unengaged Mind: Defining Boredom in Terms of Attention. Perspect. Psychol. Sci..

[84] Jackson S.A., Marsh H.W. (1996). Development and Validation of a Scale to Measure Optimal Experience: The Flow State Scale. J. Sport Exerc. Psychol..

[85] Ulrich M., Keller J., Hoenig K., Waller C., Grön G. (2014). Neural correlates of experimentally induced flow experiences. Neuroimage.

[86] Silvia P.J., Beaty R.E., Nusbaum E.C., Eddington K.M., Kwapil T.R. (2014). Creative motivation: Creative achievement predicts cardiac autonomic markers of effort during divergent thinking. Biol. Psychol..

[87] Wheeler M.S., Arnkoff D.B., Glass C.R. (2017). The Neuroscience of Mindfulness: How Mindfulness Alters the Brain and Facilitates Emotion Regulation. Mindfulness.

[88] Mayberg H.S., Lozano A.M., Voon V., McNeely H.E., Seminowicz D., Hamani C., Schwalb J.M., Kennedy S.H. (2005). Deep Brain Stimulation for Treatment-Resistant Depression. Neuron.

[89] Emons W.H., Habibović M., Pedersen S.S. (2019). Prevalence of anxiety in patients with an implantable cardioverter defibrillator: Measurement equivalence of the HADS-A and the STAI-S. Qual. Life Res..

[90] Hermans F., Blondeel A., Arents E., Calders P., Troosters T., Derom E., Demeyer H. (2023). Validity of the Fitbit Charge 4 to measure daily steps, oxygen saturation and resting heart rate in patients with COPD. Eur. Respir. J..

[91] Jachymek M., Jachymek M.T., Kiedrowicz R.M., Kaźmierczak J., Płońska-Gościniak E., Peregud-Pogorzelska M. (2021). Wristbands in Home-Based Rehabilitation—Validation of Heart Rate Measurement. Sensors.

[92] Nissen M., Slim S., Jäger K., Flaucher M., Huebner H., Danzberger N., Fasching P.A., Beckmann M.W., Gradl S., Eskofier B.M. (2022). Heart Rate Measurement Accuracy of Fitbit Charge 4 and Samsung Galaxy Watch Active2: Device Evaluation Study. JMIR Form. Res..

[93] Neacsiu A.D., Beynel L., Powers J.P., Szabo S.T., Appelbaum L.G., Lisanby S.H., LaBar K.S. (2022). Enhancing Cognitive Restructuring with Concurrent Repetitive Transcranial Magnetic Stimulation: A Transdiagnostic Randomized Controlled Trial. Psychother. Psychosom..

[94] Appelhans B.M., Luecken L.J. (2006). Heart Rate Variability as an Index of Regulated Emotional Responding. Rev. Gen. Psychol..

[95] Allen J.J.B., Chambers A.S., Towers D.N. (2007). The many metrics of cardiac chronotropy: A pragmatic primer and a brief comparison of metrics. Biol. Psychol..

[96] Stanley J., Peake J.M., Buchheit M. (2013). Cardiac Parasympathetic Reactivation Following Exercise: Implications for Training Prescription. Sports Med..

[97] Haiblum-Itskovitch S., Czamanski-Cohen J., Galili G. (2018). Emotional Response and Changes in Heart Rate Variability Following Art-Making With Three Different Art Materials. Front. Psychol..

[98] (2024). Fitabase [Computer software]. https://www.fitabase.com/.

[99] Farokhi M. (2011). Art Therapy In Humanistic Psychiatry. Proced. Soc. Behav. Sci..

[100] Spielberger C.D. (2012). State-Trait Anxiety Inventory for Adults. [Dataset].

[101] Lovibond S.H., Lovibond P.F. (1995).

[102] Brown K.W., Ryan R.M. (2003). The benefits of being present: Mindfulness and its role in psychological well-being. J. Pers. Soc. Psychol..

[103] Costa P.T., McCrae R.R. (1992).

[104] Grove J.R., Prapavessis H. (1992). Preliminary evidence for the reliability and validity of an abbreviated Profile of Mood States. Int. J. Sport Psychol..

[105] Wolpe J. (1969). The practice of behavior therapy.

[106] Tse D.C.K., Nakamura J., Csikszentmihalyi M. (2021). Living well by “flowing’ well: The indirect effect of autotelic personality on well-being through flow experience. J. Posit. Psychol..

