# Supplementary material for: Selective emotion regulation in creative art production: Psychophysiological reactivity during painting reduces anxiety
Source: iScience. 2025 Apr 28;28(6):112543. doi: 10.1016/j.isci.2025.112543 (PMC12225899; doi:10.1016/j.isci.2025.112543)
Supplement: Document S1. Figures S1–S18, Table S1, Methods S1–S3, and Data S1–S18 [file mmc1.pdf]

iScience, Volume *28*

Supplemental information

Selective emotion regulation in creative art production: Psychophysiological reactivity during painting reduces anxiety

Lucas Bellaiche, Kayla Lihardo, Chloe Williams, Jill Chaffee, Kevin S. LaBar, and Paul Seli

Method S1: Behavioral (self-report) materials, related to STAR Methods

The Mindfulness Attention Awareness Scale (MAAS) indexed a measure of awareness of one's own behavior [S1]. Openness to experience—a common predictor for creativity (see [S2])—was collected via the NEO Five-Factor Inventory [S3]. Fluid intelligence was measured via the Culture Fair Intelligence Test [S4], in which participants are given three minutes to solve as many sequence-completion questions of 13 total questions. In each question, three figures that follow a sequential pattern is shown, and the participant must choose a fourth option from six possible solutions that accurately continues the pattern. The Inventory of Creative Activities and Achievements [S5] measured creative activities and accomplishments (modality-specific and aggregated across modalities). Measures of growth versus fixed creative mindsets were collected utilizing the Creative Mindset Scale [S6]. The trait-level portion of the State-Trait Anxiety Inventory (STAI) questionnaire was administered to measure general levels of anxiety [S7]. Measures of mind-wandering varying in spontaneity and deliberateness were collected via the scale by [S8]. To measure individual's habitual use of ER strategies, we implemented the Emotion Regulation Questionnaire [S9]. Lastly, measures of negative emotional psychopathologies were collected via the 21-item Depression, Anxiety, and Stress Scale [S10]. All questionnaires were aggregated by summation with items reverse-scored when appropriate; fluid intelligence was further divided by 13 to obtain a percentage accurate score.

To assess flow state, we administered the 36-item Flow State Scale [S11], which indexes nine aspects of a flow state that aggregate into a Total Flow score: challenge-skill balance, action-awareness balance, clear goals, unambiguous feedback, concentration, sense of control, loss of self-consciousness, transformation of time, and autotelic experience.

Method S2: Physiological data validation, related to STAR Methods

Compared to an electrocardiogram Holter device (a gold-standard instrument for HR), the Fitbit Charge 4 is a relatively underutilized tool for HR measurements in psychology research but importantly demonstrates high agreement to the Holter device with a concordance correlation coefficient of .81 [S12]. In particular, the Fitbit device seems to be more accurate during sedentary activities (e.g., typing) than during active exercise activities ([S12]; [S13]). Another recent validation study of the Charge 4 during sedentary activity also demonstrated a large correlation effect size (according to interpretation guidelines from [S14] between HR from the Charge 4 and from a Polar chest strap monitor [S15]. Thus, given that our experiment design involved relatively sedentary activity (and not exercise activity), we felt confident in the accuracy of the Fitbit device in this study.

One consideration in using the Fitbit to track physiological activity is that Fitbit does not readily release the interbeat interval data, which makes it impossible to calculate heart rate variability (HRV), a well-known correlate of momentary stress, well-being, and ER (e.g., [S16]; see [S17]). Fitbit does release calculated nightly HRV after three nights of consumer wear, though this was irrelevant to us due to our study design. Nonetheless, we used basic HR as an index for physiological reactivity. While there is no mathematical way to calculate HRV from simple average HR values, it is well-accepted that the two strongly inversely correlate with one another ([S18]; [S19]); some researchers further suggest that “HRV is dependent on HR” [S18] (p. 1). Crucially, [S20] propose that non-metabolic (i.e., unrelated to physical activity) HR can be a reliable measure of mental states, specifically emotions. However, they also suggest non-metabolic HR to be assessed in tandem with subjective ratings of emotion, while also considering recent physical activity. This study follows these guidelines.

Method S3: Physiological data quality checks, related to STAR Methods

The HR measurements recorded in the three minutes preceding each task were used as the baseline measurements. In total, across participants and conditions, five baseline values were missing: three participants each were missing one baseline data point, while one participant was missing two baseline data points. Three of these values correspond to the baseline HR preceding the painting condition (two preceding the mazes condition), and three missing baseline values correspond to Day 1 (two corresponding to Day 2). No participant was missing all three baseline data points. No data imputing was done; baseline averages were calculated from the available baseline data points recorded from the three minutes. There was no significant difference between the average baseline heart rate measurements of the painting condition ($M = 78.55$, $SD = 10.96$) and maze condition ($M = 77.95$, $SD = 13.15$), $t(97) = 0.529$, $p = .60$.

During the conditions themselves (task-related HR), 29 data points (.73%) were missing from the total expected HR data (total expected: 20 data points for each condition across 2 days per 98 participants for a total of 1960 data points). 13 participants were missing one value, five participants were missing two values, and two participants were missing three values. There were 14 missing values from the painting condition (15 from the mazes condition), and 19 missing values from Day 1 measurements (10 from Day 2). No data imputing was done; analyses of task-related HR accordingly excluded missing values. For instance, the Average HR for a condition with only 19 data points instead of 20 simply summed the 19 values and divided by 19.

To correct for baseline, each value in the task-related HR (i.e., during a condition) was divided by the average of the three minutes preceding that day's condition. By using division as our baseline-correction method (rather than subtraction), we account for differences among participants not only in their baseline HR data, but also the magnitude by which the HR changes during the task. Thus, our units of analyses are ratio scores of task-related HR (originally in bpm) as compared to that the baseline HR of that day for that participant. This meaningfully captures within-person variability and standardizes analyses and visualizations (such that baseline = 1). However, we note that the significant results from the below analyses did not differ whether we used raw or baseline-corrected HR values (see Figures S11-12, Data S10-11).

Figure S1

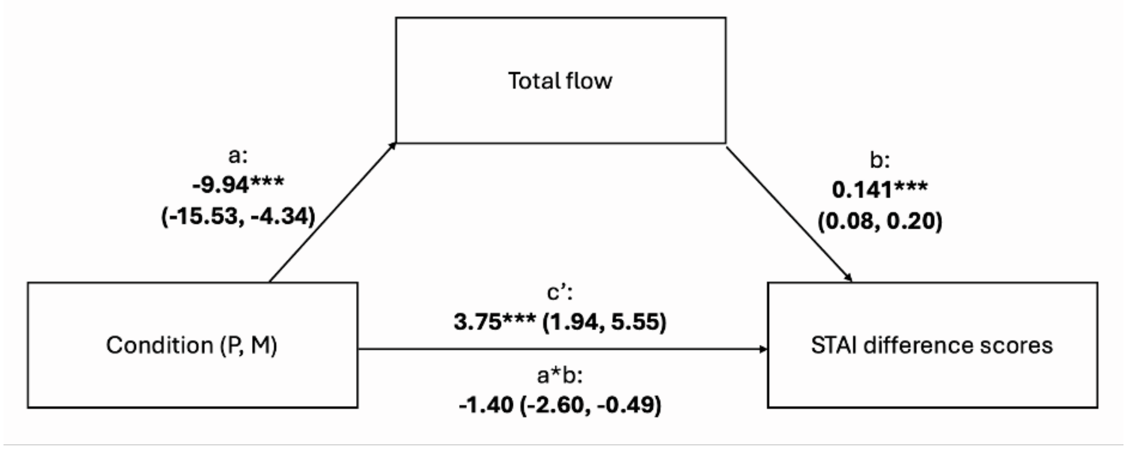

Fig. S1: Mediation model, including only Flow, related to Figure 2.

Figure S2

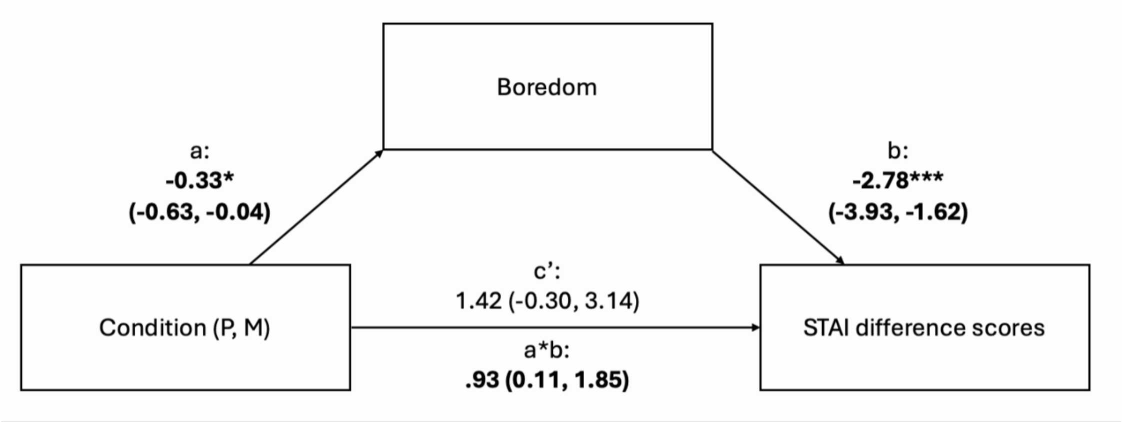

Fig. S2: Mediation model, including only Boredom, related to Figure 2.

Data S1: Demographics

```
## [1] "Mean age: 21.1818181818182"

## [1] "SD age: 4.2242343500516"

## [1] "Male count: 27"

## [1] "Female count: 72"
```

Figure S3:

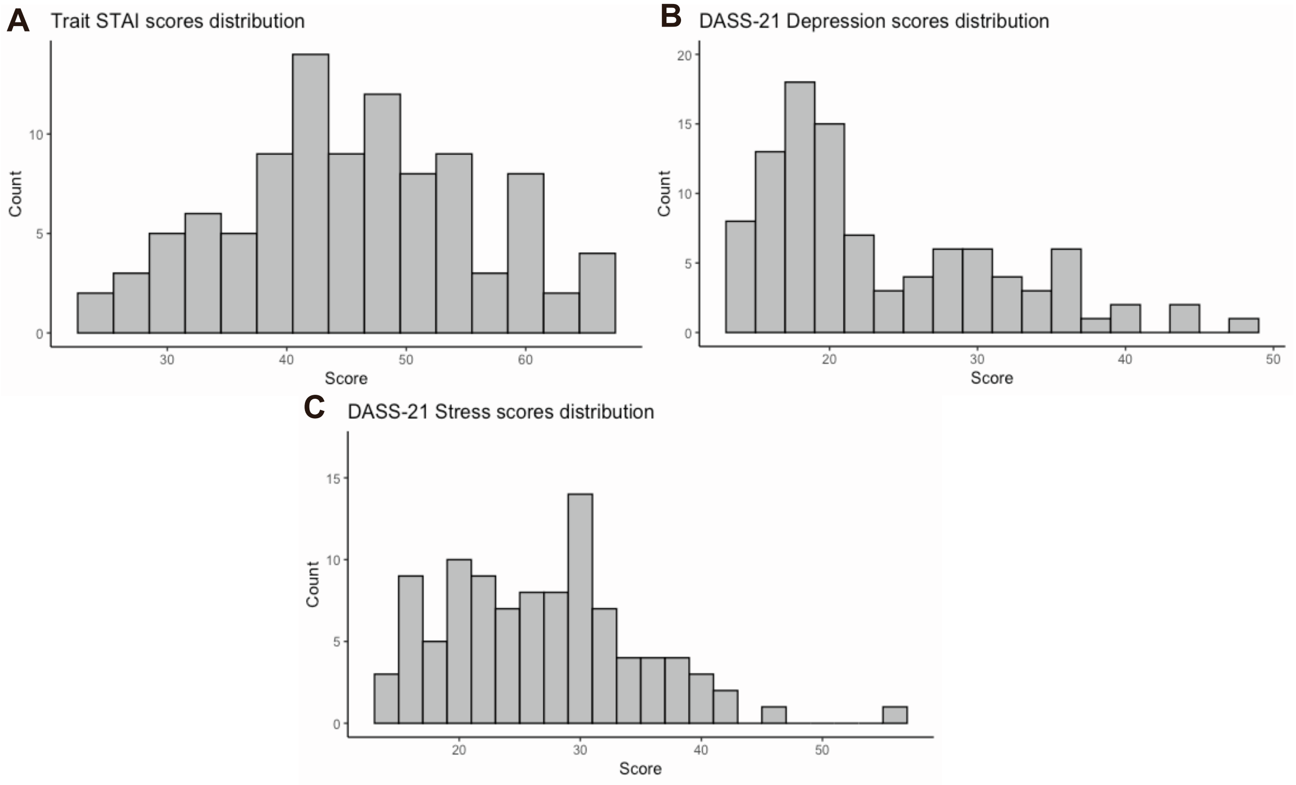

Fig S3: Trait **(A)** STAI (anxiety), **(B)** Depression, and **(C)** Stress distributions, related to STAR Methods.

Figure S4:

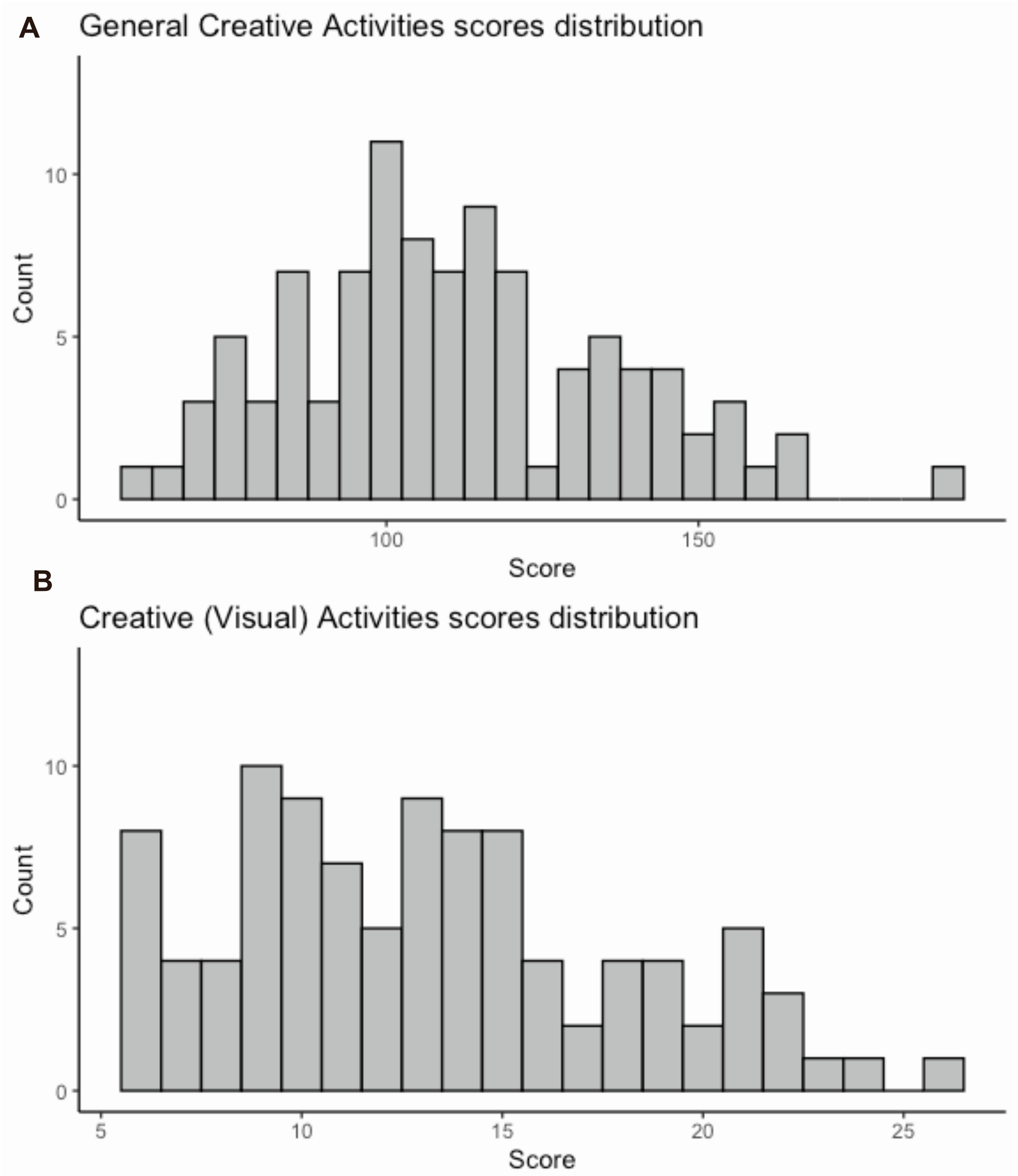

Fig. S4: Experience in **(A)** general creative activities, **(B)** visual creative activities distribution, related to STAR Methods.

Figure S5:

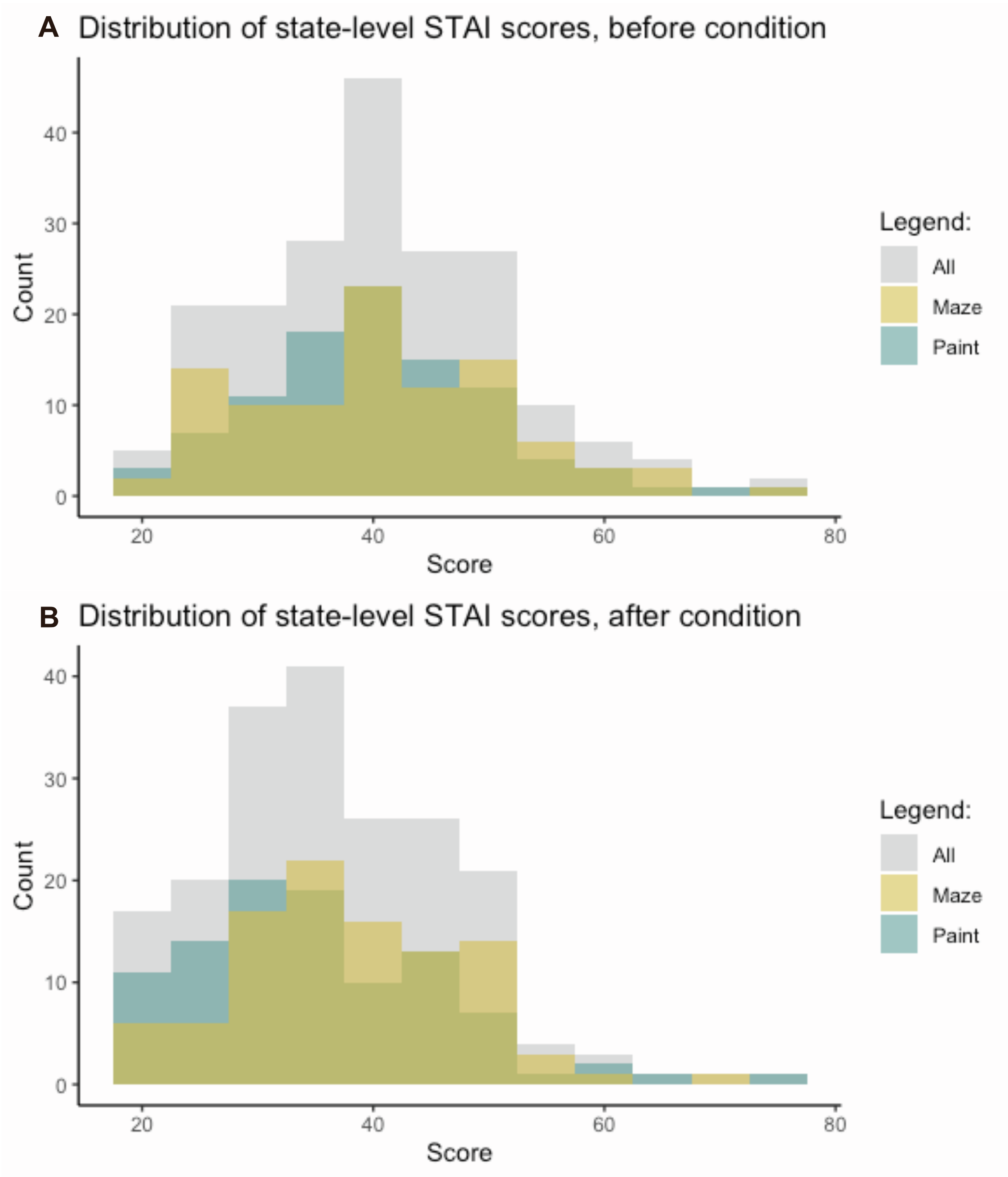

Fig. S5: Distribution of (state) STAI scores **(A)** before and **(B)** after assigned condition, collapsed across Days 1 and 2. Related to Figure 1a.

Data S2: Analyses of change in POMS, divided by Negative, Positive subscales, with plots:

POMS: negative

Effect	df	MSE	F	pes	p.value
Condition	1, 98	130.04	0.14	.001	.706
variable	1, 98	53.67	81.00 ***	.453	<.001
Condition:variable	1, 98	45.69	0.58	.006	.450

POMS: positive

Effect	df	MSE	F	pes	p.value
Condition	1, 98	41.59	0.03	<.001	.864
variable	1, 98	17.10	37.82 ***	.278	<.001
Condition:variable	1, 98	17.76	0.86	.009	.355

Figure S6:

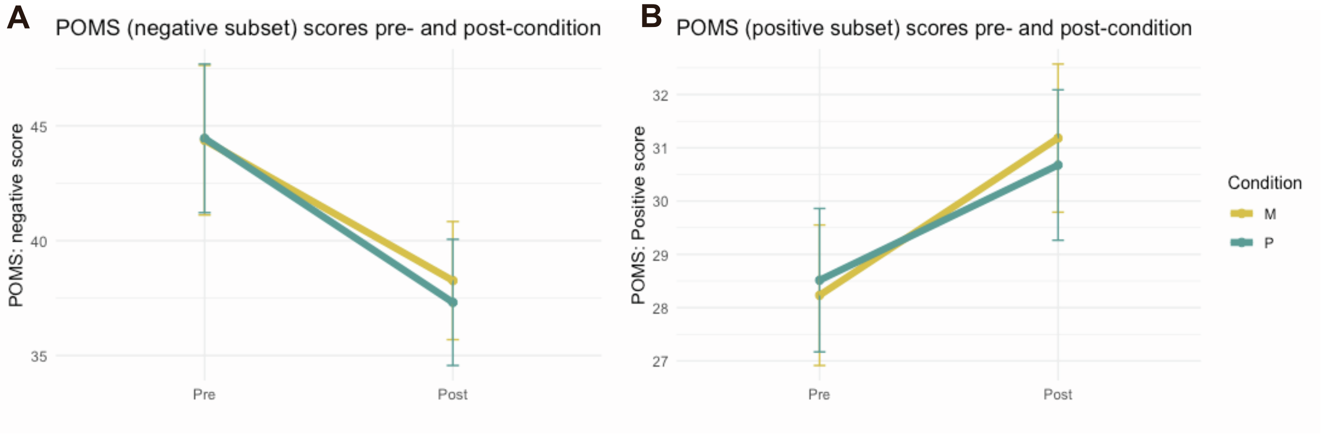

Fig. S6: Change in **(A)** negative and **(B)** positive mood before and after assigned condition, collapsed across Days 1 and 2. No significant interaction between Time (pre, post) and Condition (Paint, Maze) for either subset. Related to Figure 1c. Data are represented as mean +/- 95% confidence intervals.

Data S3: Primary self-report analyses (change in affect by condition) maintain when including Condition Order (CondOrder) as a moderator

STAI (anxiety)

Effect	df	MSE	F	pes	p.value
CondOrder	1, 97	316.49	2.00	.020	.160
Condition	1, 97	72.68	4.01 *	.040	.048
CondOrder:Condition	1, 97	72.68	1.22	.012	.272
variable	1, 97	36.45	38.12 ***	.282	<.001
CondOrder:variable	1, 97	36.45	0.02	<.001	.891
Condition:variable	1, 97	20.83	5.94 *	.058	.017
CondOrder:Condition:variable	1, 97	20.83	2.52	.025	.116

SUDS (mental distress)

Effect	df	MSE	F	pes	p.value
CondOrder	1, 97	1140.63	0.99	.010	.323
Condition	1, 97	232.40	0.46	.005	.501
CondOrder:Condition	1, 97	232.40	0.15	.002	.703
variable	1, 97	62.51	52.61 ***	.352	<.001
CondOrder:variable	1, 97	62.51	0.58	.006	.449
Condition:variable	1, 97	61.35	0.46	.005	.499
CondOrder:Condition:variable	1, 97	61.35	1.18	.012	.281

POMS (mood disturbance)

Effect	df	MSE	F	pes	p.value
CondOrder	1, 97	821.35	2.62	.026	.109
Condition	1, 97	224.83	0.04	<.001	.847
CondOrder:Condition	1, 97	224.83	4.53 *	.045	.036
variable	1, 97	89.58	93.40 ***	.491	<.001
CondOrder:variable	1, 97	89.58	1.47	.015	.228
Condition:variable	1, 97	68.58	0.04	<.001	.847
CondOrder:Condition:variable	1, 97	68.58	21.96 ***	.185	<.001

POMS: neg

Effect	df	MSE	F	pes	p.value
CondOrder	1, 97	650.38	1.85	.019	.177
Condition	1, 97	114.59	0.13	.001	.715
CondOrder:Condition	1, 97	114.59	14.22 ***	.128	<.001
variable	1, 97	53.74	81.04 ***	.455	<.001
CondOrder:variable	1, 97	53.74	0.86	.009	.356
Condition:variable	1, 97	33.80	0.89	.009	.349
CondOrder:Condition:variable	1, 97	33.80	35.47 ***	.268	<.001

POMS: pos

Effect	df	MSE	F	pes	p.value
CondOrder	1, 97	111.25	1.24	.013	.269
Condition	1, 97	41.29	0.03	<.001	.874
CondOrder:Condition	1, 97	41.29	1.72	.017	.193
variable	1, 97	17.05	38.07 ***	.282	<.001
CondOrder:variable	1, 97	17.05	1.28	.013	.260
Condition:variable	1, 97	17.76	0.85	.009	.360
CondOrder:Condition:variable	1, 97	17.76	0.98	.010	.324

Data S4: NextDay Analyses: How does change in affect extend into the next day (Timepoint 3)?

STAI

Effect	df	MSE	F	pes	p.value
Condition	1, 96	46.63	0.76	.008	.384
variable	2, 192	34.45	26.41 ***	.216	<.001
Condition:variable	2, 192	42.30	6.05 **	.059	.003

```
## Condition_pairwise variable_pairwise estimate SE df t.ratio p.value
## M - P pre_STAI - post_STAI -2.06 0.937 96 -2.201 0.0876
## M - P pre_STAI - nextday_STAI 2.53 1.453 96 1.738 0.2348
## M - P post_STAI - nextday_STAI 4.59 1.498 96 3.062 0.0085

## variable_pairwise estimate SE df t.ratio p.value
## pre_STAI - post_STAI 3.7938 0.615 96 6.169 <.0001
## pre_STAI - nextday_STAI 0.0876 0.502 96 0.175 0.9833
## post_STAI - nextday_STAI -3.7062 0.660 96 -5.616 <.0001

## Condition = M:
## variable_pairwise estimate SE df t.ratio p.value
## pre_STAI - post_STAI 2.76 0.809 96 3.414 0.0027
## pre_STAI - nextday_STAI 1.35 0.844 96 1.599 0.2509
## post_STAI - nextday_STAI -1.41 0.927 96 -1.523 0.2847
##
## Condition = P:
## variable_pairwise estimate SE df t.ratio p.value
## pre_STAI - post_STAI 4.82 0.735 96 6.565 <.0001
## pre_STAI - nextday_STAI -1.18 0.920 96 -1.278 0.4112
## post_STAI - nextday_STAI -6.00 1.065 96 -5.636 <.0001

## variable = pre_STAI:
## Condition_pairwise estimate SE df t.ratio p.value
## M - P 0.649 0.895 96 0.725 0.4699
##
## variable = post_STAI:
## Condition_pairwise estimate SE df t.ratio p.value
## M - P 2.711 1.074 96 2.524 0.0133
##
## variable = nextday_STAI:
## Condition_pairwise estimate SE df t.ratio p.value
## M - P -1.876 0.866 96 -2.166 0.0328
```

SUDS

Effect	df	MSE	F	pes	p.value
Condition	1, 96	142.59	0.16	.002	.686
variable	2, 192	78.10	24.67 ***	.204	<.001
Condition:variable	2, 192	125.62	0.50	.005	.609

```
## variable_pairwise estimate SE df t.ratio p.value
## pre_SUDS - post_SUDS 5.76 0.807 96 7.136 <.0001
## pre_SUDS - nextday_SUDS 0.66 0.789 96 0.836 0.6818
## post_SUDS - nextday_SUDS -5.10 1.068 96 -4.772 <.0001

## variable emmean SE df lower.CL upper.CL
## pre_SUDS 27.3 1.82 96 23.6 30.9
## post_SUDS 21.5 1.73 96 18.1 24.9
## nextday_SUDS 26.6 1.96 96 22.7 30.5
```

POMS

Effect	df	MSE	F	pes	p.value
Condition	1, 96	150.37	0.21	.002	.649
variable	2, 192	87.99	47.85 ***	.333	<.001
Condition:variable	2, 192	121.70	0.71	.007	.494

```
##  variable_pairwise       estimate    SE df t.ratio p.value
##  pre_POMS - post_POMS         9.25 0.961 96   9.624  <.0001
##  pre_POMS - nextday_POMS      3.68 0.883 96   4.166  0.0002
##  post_POMS - nextday_POMS    -5.57 1.008 96  -5.527  <.0001
##
## Results are averaged over the levels of: Condition
## P value adjustment: tukey method for comparing a family of 3 estimates

##  variable     emmean   SE df lower.CL upper.CL
##  pre_POMS      16.03 1.68 96    12.70     19.4
##  post_POMS      6.78 1.42 96     3.95      9.6
##  nextday_POMS  12.35 1.61 96     9.15     15.6
##
## Results are averaged over the levels of: Condition
## Confidence level used: 0.95
```

Figure S7:

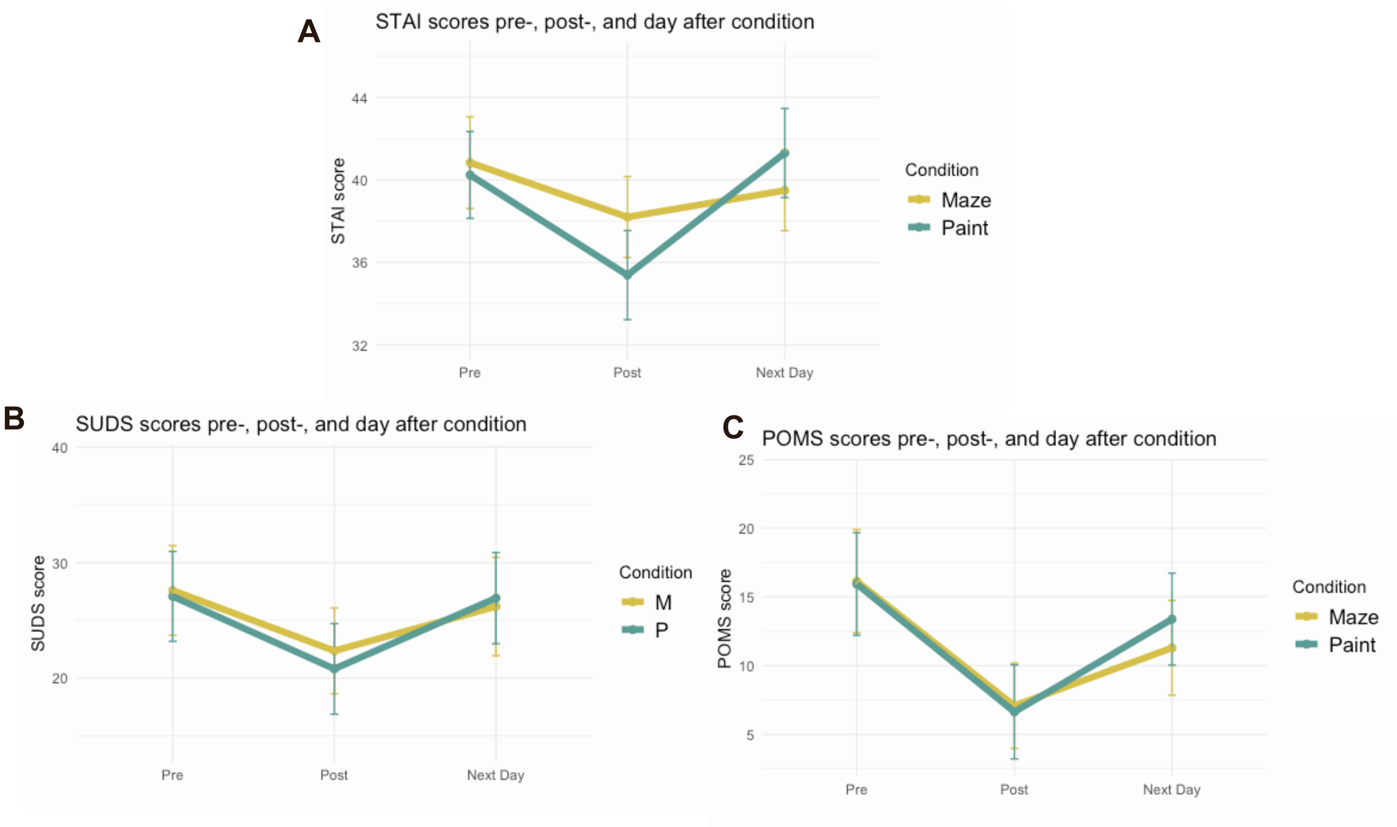

Fig. S7: (A) STAI, **(B)** SUDS, and **(C)** POMS scores before condition, after condition, and at the next day (before a new condition, if on Day 2). A significant decrease (across conditions) emerged between Pre and Next Day for POMS only, $p < .001$. Related to Figure 1. Data are represented as mean +/- 95% confidence intervals.

Data S5: NextDay results maintain when including Condition Order (CondOrder) as a moderator

STAI

Effect	df	MSE	F	pes	p.value
CondOrder	1, 95	466.75	1.51	.016	.222
Condition	1, 95	46.66	0.78	.008	.379
CondOrder:Condition	1, 95	46.66	0.94	.010	.336
variable	2, 190	34.50	26.31 ***	.217	<.001
CondOrder:variable	2, 190	34.50	0.87	.009	.423
Condition:variable	2, 190	42.01	6.16 **	.061	.003
CondOrder:Condition:variable	2, 190	42.01	1.65	.017	.194

SUDS

Effect	df	MSE	F	pes	p.value
CondOrder	1, 95	1814.04	1.11	.012	.294
Condition	1, 95	143.97	0.16	.002	.689
CondOrder:Condition	1, 95	143.97	0.08	<.001	.783
variable	2, 190	78.76	24.50 ***	.205	<.001
CondOrder:variable	2, 190	78.76	0.19	.002	.826
Condition:variable	2, 190	126.59	0.49	.005	.611
CondOrder:Condition:variable	2, 190	126.59	0.26	.003	.769

POMS

Effect	df	MSE	F	pes	p.value
CondOrder	1, 95	1253.12	2.17	.022	.144
Condition	1, 95	144.99	0.24	.002	.628
CondOrder:Condition	1, 95	144.99	4.56 *	.046	.035
variable	2, 190	88.34	47.75 ***	.334	<.001
CondOrder:variable	2, 190	88.34	0.62	.006	.539
Condition:variable	2, 190	113.51	0.74	.008	.479
CondOrder:Condition:variable	2, 190	113.51	7.93 ***	.077	<.001

Data S6: Primary ANCOVAs for affect: Change-in-affect results maintain when controlling for recent Exercise, Caffeine, and Substance intake

STAI

Effect	df	MSE	F	ges	p.value
Paint_Exercise	1, 92	308.10	1.54	.011	.218
Paint_Caffeine	1, 92	308.10	0.10	<.001	.753
Paint_Substances	1, 92	308.10	3.52 +	.025	.064
Maze_Exercise	1, 92	308.10	0.33	.002	.566
Maze_Caffeine	1, 92	308.10	0.68	.005	.411
Maze_Substances	1, 92	308.10	2.61	.018	.110
Condition	1, 92	74.15	5.20 *	.009	.025
Paint_Exercise:Condition	1, 92	74.15	0.01	<.001	.928
Paint_Caffeine:Condition	1, 92	74.15	0.43	<.001	.514
Paint_Substances:Condition	1, 92	74.15	0.13	<.001	.724
Maze_Exercise:Condition	1, 92	74.15	0.67	.001	.415
Maze_Caffeine:Condition	1, 92	74.15	0.01	<.001	.913
Maze_Substances:Condition	1, 92	74.15	0.75	.001	.390
variable	1, 92	36.76	33.66 ***	.028	<.001
Paint_Exercise:variable	1, 92	36.76	2.39	.002	.126
Paint_Caffeine:variable	1, 92	36.76	0.56	<.001	.455
Paint_Substances:variable	1, 92	36.76	0.20	<.001	.659
Maze_Exercise:variable	1, 92	36.76	1.42	.001	.236
Maze_Caffeine:variable	1, 92	36.76	0.05	<.001	.832
Maze_Substances:variable	1, 92	36.76	0.47	<.001	.496
Condition:variable	1, 92	22.02	5.54 *	.003	.021
Paint_Exercise:Condition:variable	1, 92	22.02	0.65	<.001	.422
Paint_Caffeine:Condition:variable	1, 92	22.02	0.00	<.001	.967
Paint_Substances:Condition:variable	1, 92	22.02	0.07	<.001	.785
Maze_Exercise:Condition:variable	1, 92	22.02	0.16	<.001	.688
Maze_Caffeine:Condition:variable	1, 92	22.02	0.05	<.001	.829
Maze_Substances:Condition:variable	1, 92	22.02	0.80	<.001	.374

SUDS

Effect	df	MSE	F	ges	p.value
Paint_Exercise	1, 92	1133.69	0.09	<.001	.764
Paint_Caffeine	1, 92	1133.69	0.03	<.001	.859
Paint_Substances	1, 92	1133.69	3.73 +	.029	.056
Maze_Exercise	1, 92	1133.69	0.09	<.001	.764
Maze_Caffeine	1, 92	1133.69	0.55	.004	.460
Maze_Substances	1, 92	1133.69	3.34 +	.026	.071
Condition	1, 92	229.06	1.12	.002	.292
Paint_Exercise:Condition	1, 92	229.06	0.13	<.001	.717

Effect	df	MSE	F	ges	p.value
Paint_Caffeine:Condition	1, 92	229.06	1.72	.003	.193
Paint_Substances:Condition	1, 92	229.06	0.01	<.001	.918
Maze_Exercise:Condition	1, 92	229.06	0.46	<.001	.501
Maze_Caffeine:Condition	1, 92	229.06	0.26	<.001	.609
Maze_Substances:Condition	1, 92	229.06	2.63	.004	.108
variable	1, 92	65.39	44.54 ***	.019	<.001
Paint_Exercise:variable	1, 92	65.39	0.08	<.001	.781
Paint_Caffeine:variable	1, 92	65.39	0.08	<.001	.779
Paint_Substances:variable	1, 92	65.39	0.05	<.001	.830
Maze_Exercise:variable	1, 92	65.39	0.40	<.001	.531
Maze_Caffeine:variable	1, 92	65.39	0.17	<.001	.680
Maze_Substances:variable	1, 92	65.39	0.07	<.001	.796
Condition:variable	1, 92	60.94	1.19	<.001	.277
Paint_Exercise:Condition:variable	1, 92	60.94	0.22	<.001	.640
Paint_Caffeine:Condition:variable	1, 92	60.94	2.38	<.001	.127
Paint_Substances:Condition:variable	1, 92	60.94	1.11	<.001	.294
Maze_Exercise:Condition:variable	1, 92	60.94	0.01	<.001	.912
Maze_Caffeine:Condition:variable	1, 92	60.94	2.64	.001	.107
Maze_Substances:Condition:variable	1, 92	60.94	0.02	<.001	.888

POMS

Effect	df	MSE	F	ges	p.value
Paint_Exercise	1, 92	803.95	2.46	.017	.120
Paint_Caffeine	1, 92	803.95	0.21	.001	.649
Paint_Substances	1, 92	803.95	2.06	.014	.155
Maze_Exercise	1, 92	803.95	0.63	.004	.429
Maze_Caffeine	1, 92	803.95	0.46	.003	.498
Maze_Substances	1, 92	803.95	1.05	.007	.308
Condition	1, 92	219.93	0.90	.002	.345
Paint_Exercise:Condition	1, 92	219.93	0.03	<.001	.858
Paint_Caffeine:Condition	1, 92	219.93	0.08	<.001	.772
Paint_Substances:Condition	1, 92	219.93	0.59	.001	.444
Maze_Exercise:Condition	1, 92	219.93	3.84 +	.007	.053
Maze_Caffeine:Condition	1, 92	219.93	0.01	<.001	.938
Maze_Substances:Condition	1, 92	219.93	1.79	.003	.185
variable	1, 92	91.77	78.97 ***	.058	<.001
Paint_Exercise:variable	1, 92	91.77	2.37	.002	.127
Paint_Caffeine:variable	1, 92	91.77	0.27	<.001	.602
Paint_Substances:variable	1, 92	91.77	0.91	<.001	.343
Maze_Exercise:variable	1, 92	91.77	0.12	<.001	.727
Maze_Caffeine:variable	1, 92	91.77	0.03	<.001	.855

Effect	df	MSE	F	ges	p.value
Maze_Substances:variable	1, 92	91.77	1.24	<.001	.268
Condition:variable	1, 92	83.11	0.00	<.001	.991
Paint_Exercise:Condition:variable	1, 92	83.11	0.33	<.001	.569
Paint_Caffeine:Condition:variable	1, 92	83.11	4.69 *	.003	.033
Paint_Substances:Condition:variable	1, 92	83.11	0.40	<.001	.526
Maze_Exercise:Condition:variable	1, 92	83.11	0.20	<.001	.655
Maze_Caffeine:Condition:variable	1, 92	83.11	0.76	<.001	.386
Maze_Substances:Condition:variable	1, 92	83.11	0.34	<.001	.563

Figure S8:

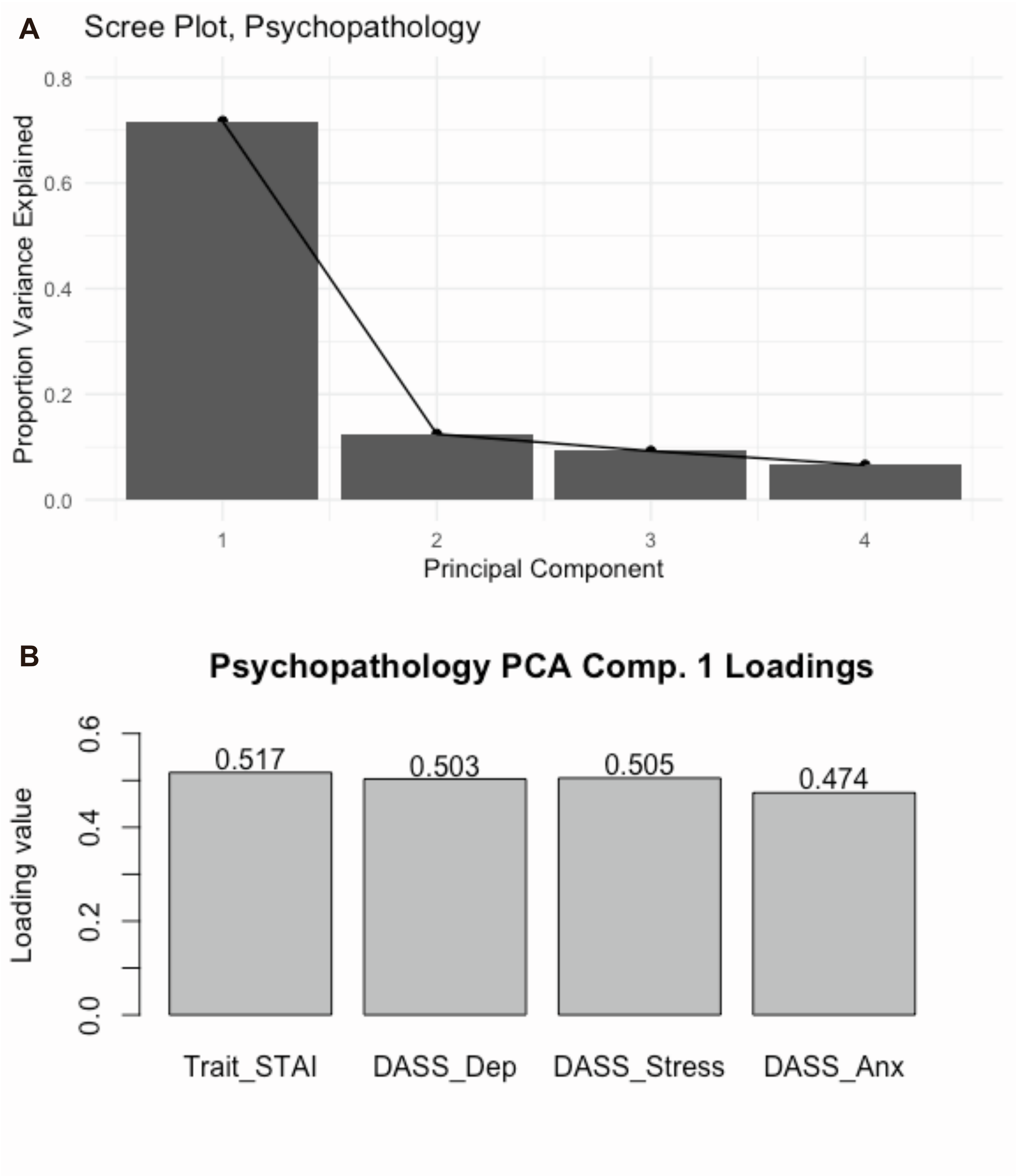

Fig. S8: (A) Scree plot and **(B)** component loadings of PCA performed on Psychopathology variables. Related to STAR Methods.

Figure S9:

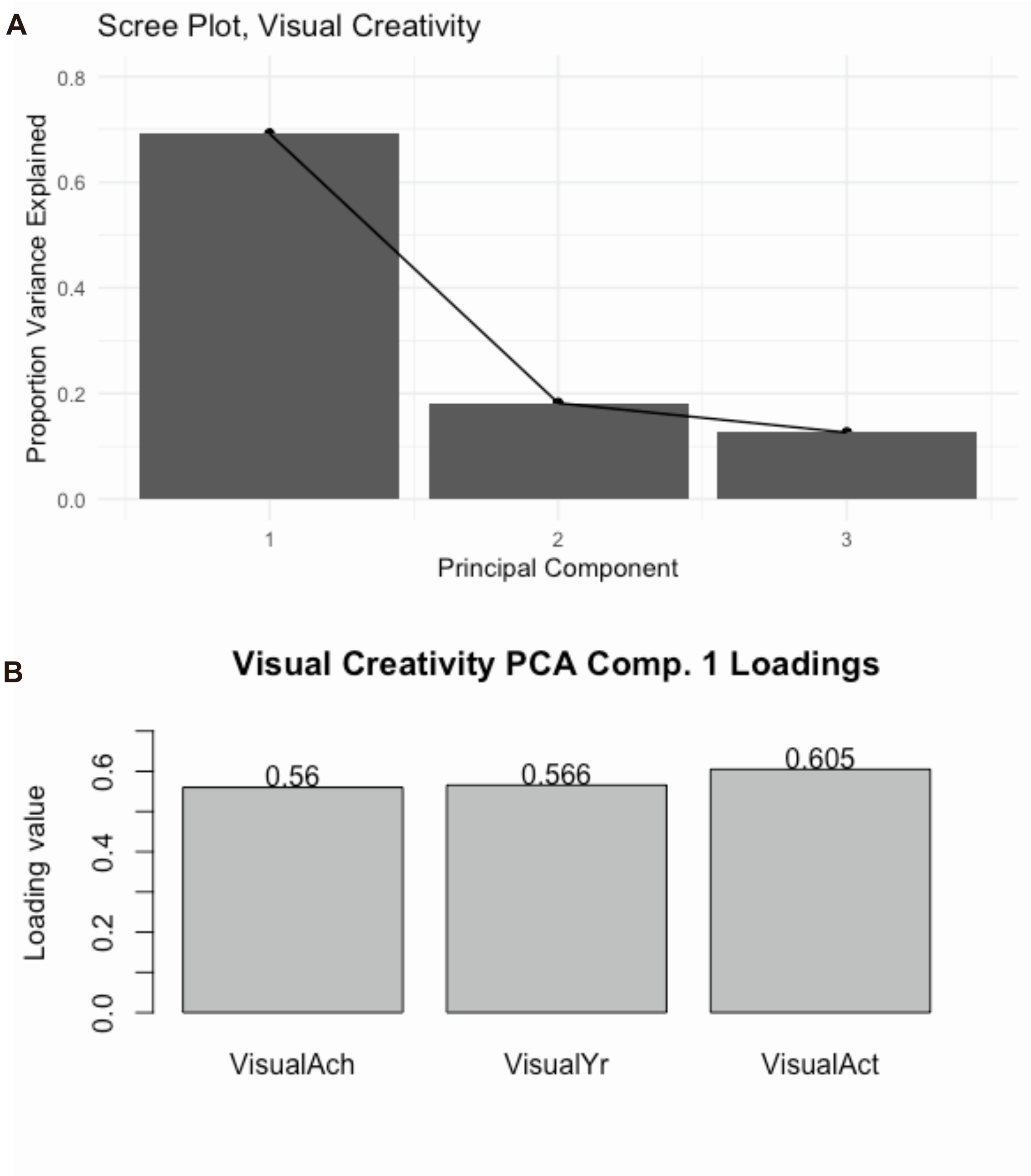

Fig. S9: (A) Scree plot and **(B)** component loadings of PCA performed on Visual Creativity variables. Related to STAR Methods.

Figure S10:

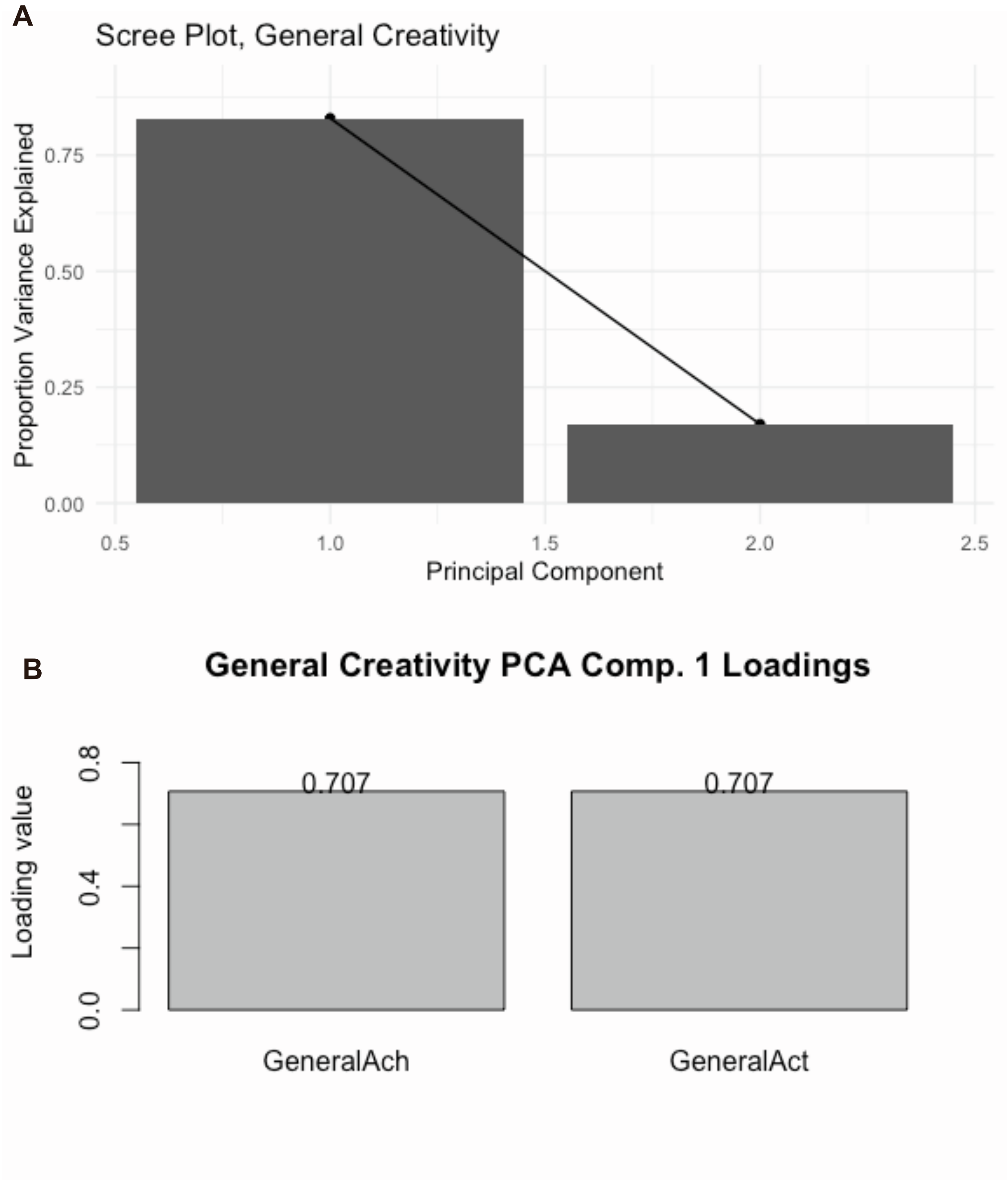

Fig. S10: (A) Scree plot and **(B)** component loadings of PCA performed on General Creativity variables. Related to STAR Methods.

Data S7: ANOVA results from PCA trait-level moderations on STAI reduction: Psychopathology and Creativity

In the ANOVA including the Psychopathology variable as a centered, between-subjects factor, we found that the interaction between Time and Condition maintained, $F(1, 97) = 5.72$, corrected $p = .038$, $\eta_p^2 = .056$, but Psychopathology did not significantly moderate the main effect of Time, $F(1, 97) = 3.57$, corrected $p = .124$, $\eta_p^2 = .035$, the main effect of Condition, $F(1, 97) = 3.34$, corrected $p = .142$, $\eta_p^2 = .033$, nor the interaction between Time and Condition, $F(1, 97) = 0.05$, corrected $p = 1$, $\eta_p^2 < .001$. Thus, trait psychopathology scores did not meaningfully moderate differences in STAI scores.

In the ANOVA including the General Creativity and Visual Creativity variables as centered, between-subjects factors, we found that the interaction between Time and Condition maintained, $F(1, 95) = 9.23$, corrected $p = .006$, $\eta_p^2 = .089$. General Creativity did not significantly moderate the main effect of Time, $F(1, 95) = 0.66$, corrected $p = .84$, $\eta_p^2 = .007$, the main effect of Condition, $F(1, 95) = 1.59$, corrected $p = .422$, $\eta_p^2 = .016$, nor the interaction between Time and Condition, $F(1, 95) = 1.83$, corrected $p = .358$, $\eta_p^2 = .019$. Similarly, Visual Creativity did not significantly moderate the main effect of Time, $F(1, 95) = 0.26$, corrected $p = 1$, $\eta_p^2 = .003$, the main effect of Condition, $F(1, 95) = 0.02$, corrected $p = 1$, $\eta_p^2 < .001$, nor the interaction between Time and Condition, $F(1, 95) = 0.08$, corrected $p = 1$, $\eta_p^2 < .001$. Lastly the four-way interaction between General Creativity, Visual Creativity, Time, and Condition was also non-significant, $F(1, 95) = 3.69$, corrected $p = .116$, $\eta_p^2 = .037$. Thus, even when controlling for each other's effect, neither visual creative nor general creative expertise significantly moderated differences in STAI scores.

Data S8: Motor differences between conditions: Comparison of calories exerted

```
##
##  Paired t-test
##
## data:  paint$Calories and maze$Calories
## t = -0.74065, df = 97, p-value = 0.4607
## alternative hypothesis: true mean difference is not equal to 0
## 95 percent confidence interval:
##  -175.98730   80.33424
## sample estimates:
## mean difference
##       -47.82653
```

Data S9: Primary ANCOVAs for Physio: Original physiological results maintain when controlling for recent Exercise, Caffeine, and Substance intake

Average HR

Effect	df	MSE	F	ges	p.value
Paint_Exercise	1, 91	0.00	1.55	.008	.216
Paint_Caffeine	1, 91	0.00	3.21 +	.017	.076
Paint_Substances	1, 91	0.00	0.01	<.001	.916
Maze_Exercise	1, 91	0.00	0.02	<.001	.892
Maze_Caffeine	1, 91	0.00	0.38	.002	.539
Maze_Substances	1, 91	0.00	0.01	<.001	.924
Condition	1, 91	0.00	12.35 ***	.058	<.001
Paint_Exercise:Condition	1, 91	0.00	1.32	.007	.254
Paint_Caffeine:Condition	1, 91	0.00	1.72	.009	.193
Paint_Substances:Condition	1, 91	0.00	0.05	<.001	.830
Maze_Exercise:Condition	1, 91	0.00	0.09	<.001	.763
Maze_Caffeine:Condition	1, 91	0.00	0.65	.003	.423
Maze_Substances:Condition	1, 91	0.00	0.46	.002	.500

Peak-to-trough

Effect	df	MSE	F	ges	p.value
Paint_Exercise	1, 91	0.01	0.00	<.001	.965
Paint_Caffeine	1, 91	0.01	0.04	<.001	.844
Paint_Substances	1, 91	0.01	0.34	.002	.560
Maze_Exercise	1, 91	0.01	0.66	.004	.418
Maze_Caffeine	1, 91	0.01	0.42	.003	.517
Maze_Substances	1, 91	0.01	0.00	<.001	.997
Condition	1, 91	0.00	14.01 ***	.051	<.001
Paint_Exercise:Condition	1, 91	0.00	0.53	.002	.467
Paint_Caffeine:Condition	1, 91	0.00	0.17	<.001	.684
Paint_Substances:Condition	1, 91	0.00	8.42 **	.032	.005
Maze_Exercise:Condition	1, 91	0.00	0.26	.001	.608
Maze_Caffeine:Condition	1, 91	0.00	0.09	<.001	.762
Maze_Substances:Condition	1, 91	0.00	5.66 *	.022	.019

End-Beginning

Effect	df	MSE	F	ges	p.value
Paint_Exercise	1, 91	0.01	3.59 +	.019	.061
Paint_Caffeine	1, 91	0.01	0.66	.003	.418
Paint_Substances	1, 91	0.01	0.00	<.001	.950
Maze_Exercise	1, 91	0.01	1.96	.010	.165
Maze_Caffeine	1, 91	0.01	0.47	.002	.495
Maze_Substances	1, 91	0.01	0.38	.002	.537
Condition	1, 91	0.01	0.43	.002	.514
Paint_Exercise:Condition	1, 91	0.01	3.49 +	.017	.065
Paint_Caffeine:Condition	1, 91	0.01	0.33	.002	.569
Paint_Substances:Condition	1, 91	0.01	1.55	.008	.216
Maze_Exercise:Condition	1, 91	0.01	0.03	<.001	.874
Maze_Caffeine:Condition	1, 91	0.01	1.17	.006	.283
Maze_Substances:Condition	1, 91	0.01	1.88	.009	.174

MAD

Effect	df	MSE	F	ges	p.value
Paint_Exercise	1, 91	0.00	0.00	<.001	.956
Paint_Caffeine	1, 91	0.00	0.39	.003	.536
Paint_Substances	1, 91	0.00	0.35	.002	.555
Maze_Exercise	1, 91	0.00	0.96	.006	.329
Maze_Caffeine	1, 91	0.00	0.02	<.001	.902
Maze_Substances	1, 91	0.00	0.03	<.001	.873
Condition	1, 91	0.00	15.77 ***	.057	<.001
Paint_Exercise:Condition	1, 91	0.00	1.20	.005	.277
Paint_Caffeine:Condition	1, 91	0.00	0.01	<.001	.911
Paint_Substances:Condition	1, 91	0.00	4.97 *	.019	.028
Maze_Exercise:Condition	1, 91	0.00	0.00	<.001	.998
Maze_Caffeine:Condition	1, 91	0.00	0.01	<.001	.928
Maze_Substances:Condition	1, 91	0.00	3.17 +	.012	.078

Figure S11:

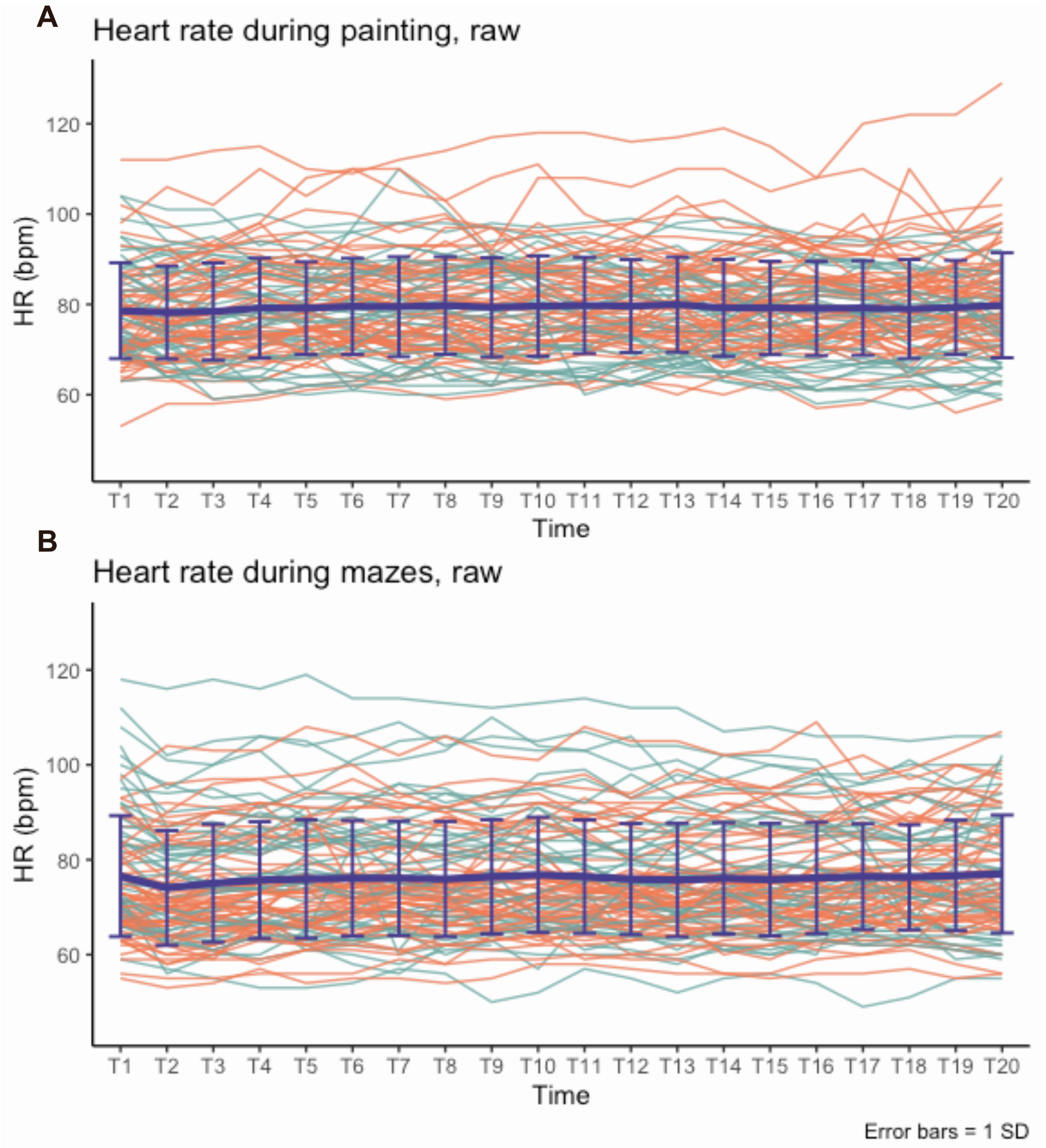

Fig. S11: Raw heart rate timeseries for **(A)** painting and **(B)** maze conditions. Blue indicates a decrease in HR across the 20 minutes, Orange indicates an increase in HR across the 20 minutes. Related to Figure 3. Error bars represent 1 standard deviation surrounding the participant mean.

Data S10: Physio results maintain when using raw (as opposed to baseline-corrected) values

Average HR

```
##
##  Paired t-test
##
## data:  paint_physio$avgHR and maze_physio$avgHR
## t = 3.615, df = 97, p-value = 0.0004785
## alternative hypothesis: true mean difference is not equal to 0
## 95 percent confidence interval:
##  1.461331 5.019369
## sample estimates:
## mean difference
##         3.24035

## [1] 0.3651726
```

End-Beginning

```
##
##  Paired t-test
##
## data:  paint_physio$range and maze_physio$range
## t = 0.74045, df = 96, p-value = 0.4608
## alternative hypothesis: true mean difference is not equal to 0
## 95 percent confidence interval:
##  -1.316904  2.883914
## sample estimates:
## mean difference
##       0.7835052

## [1] 0.07518119
```

Peak-to-trough

```
##
##  Paired t-test
##
## data:  paint_physio$diff and maze_physio$diff
## t = 3.1499, df = 97, p-value = 0.002172
## alternative hypothesis: true mean difference is not equal to 0
## 95 percent confidence interval:
##  0.8077412 3.5596058
## sample estimates:
## mean difference
##        2.183673

## [1] 0.3181834
```

MAD

```
##
##  Paired t-test
## data:  raw_physio_MAD_paint$MAD and raw_physio_MAD_maze$MAD
## t = 3.7203, df = 97, p-value = 0.0003335
## alternative hypothesis: true mean difference is not equal to 0
## 95 percent confidence interval:
##  0.2413464 0.7933355
## sample estimates:
## mean difference
##        0.517341

## [1] 0.3758057
```

Figure S12:

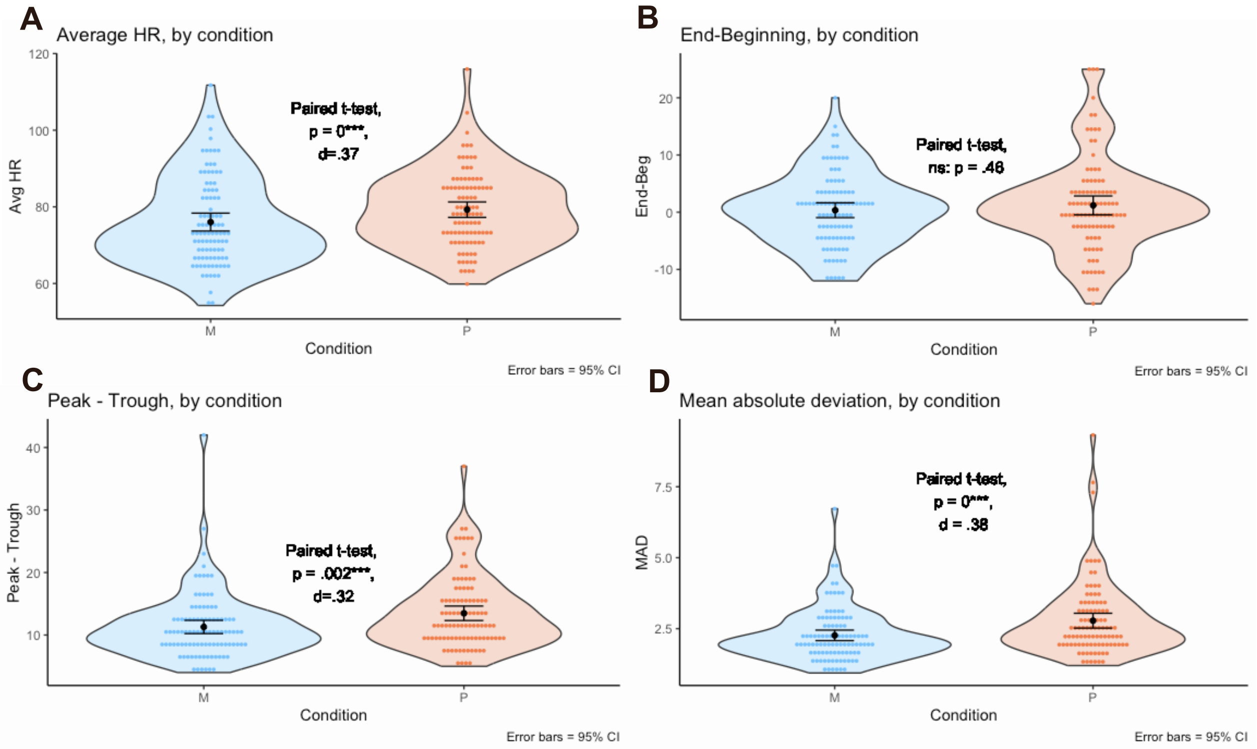

Fig. S12: (A) Higher average heart rate (raw), **(C)** higher peak-to-trough of heart rate (raw), and **(D)** higher mean absolute deviation of heart rate (raw). **(B)** No significant difference in end minus beginning heart rate (raw) values by condition. Related to Figure 3. Data are represented as mean +/- 95% confidence intervals.

Data S11: Physio results maintain when including Condition Order (CondOrder) as a moderator

Average HR

```
##                Df Sum Sq Mean Sq F value   Pr(>F)
## Condition       1 0.0601 0.06013  12.910 0.000415 ***
## Day             1 0.0131 0.01306   2.804 0.095646 .
## Condition:Day   1 0.0000 0.00001   0.003 0.959548
## Residuals     192 0.8942 0.00466
## ---
## Signif. codes:  0 '***' 0.001 '**' 0.01 '*' 0.05 '.' 0.1 ' ' 1
```

End-Beginning

```
##                Df Sum Sq Mean Sq F value Pr(>F)
## Condition       1 0.0076 0.00760   0.817  0.367
## Day             1 0.0462 0.04621   4.965  0.027 *
## Condition:Day   1 0.0012 0.00120   0.129  0.720
## Residuals     192 1.7870 0.00931
## ---
## Signif. codes:  0 '***' 0.001 '**' 0.01 '*' 0.05 '.' 0.1 ' ' 1
```

Peak-to-trough

```
##                Df Sum Sq Mean Sq F value  Pr(>F)
## Condition       1 0.0379 0.03789   7.899 0.00546 **
## Day             1 0.0119 0.01194   2.488 0.11633
## Condition:Day   1 0.0189 0.01886   3.931 0.04883 *
## Residuals     192 0.9211 0.00480
## ---
## Signif. codes:  0 '***' 0.001 '**' 0.01 '*' 0.05 '.' 0.1 ' ' 1
```

MAD

```
##                Df  Sum Sq   Mean Sq F value  Pr(>F)
## Condition       1 0.00214 0.0021429   9.910 0.00191 **
## Day             1 0.00033 0.0003253   1.505 0.22148
## Condition:Day   1 0.00126 0.0012637   5.844 0.01656 *
## Residuals     192 0.04152 0.0002162
## ---
## Signif. codes:  0 '***' 0.001 '**' 0.01 '*' 0.05 '.' 0.1 ' ' 1
```

Figure S13:

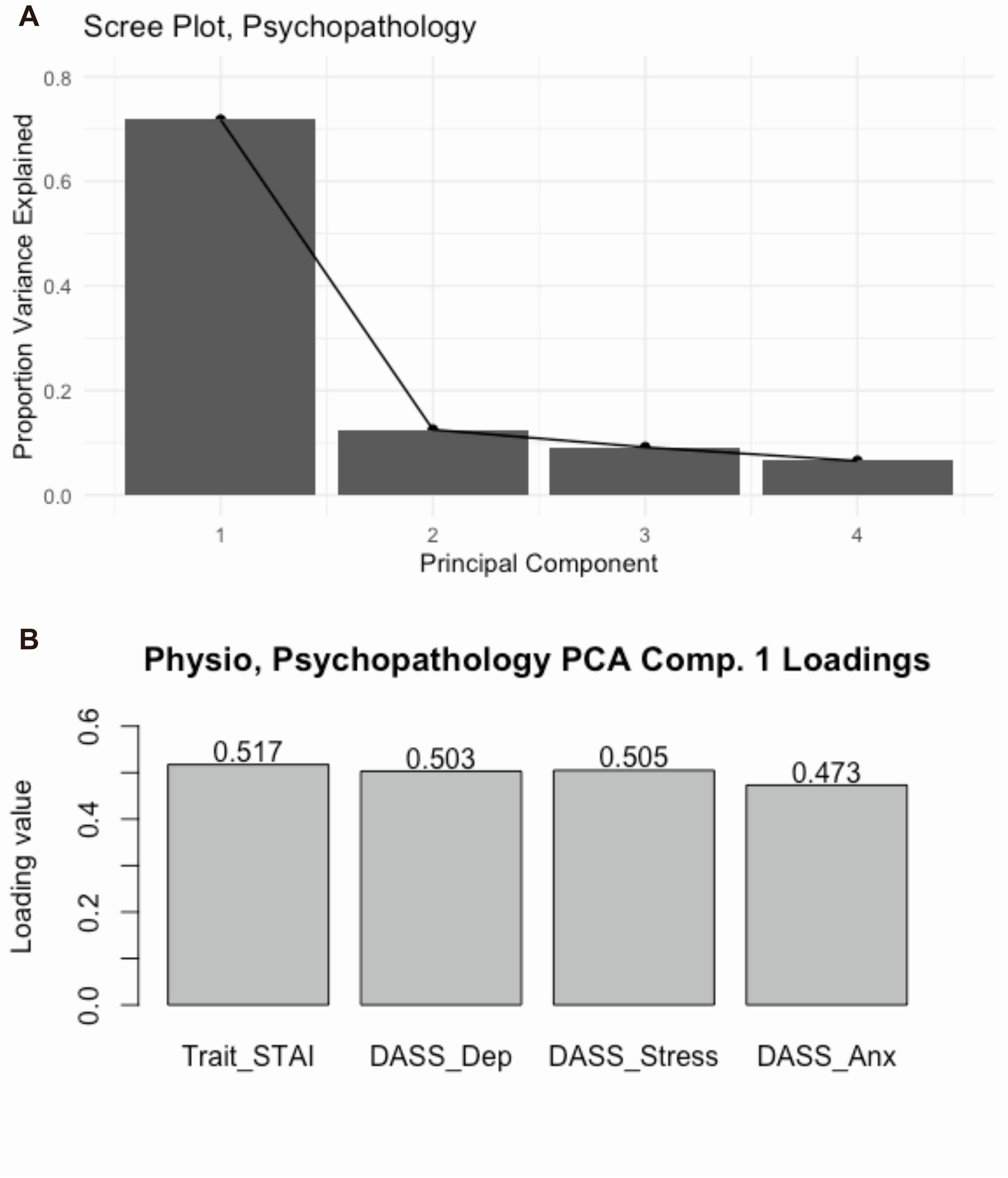

Fig. S13: Earlier PCA (Figure S8) included 99 participants. Here we show the **(A)** Scree plot and **(B)** component loadings of PCA performed on Psychopathology variables using only the 98 participants with valid physio data. Related to STAR Methods.

Figure S14:

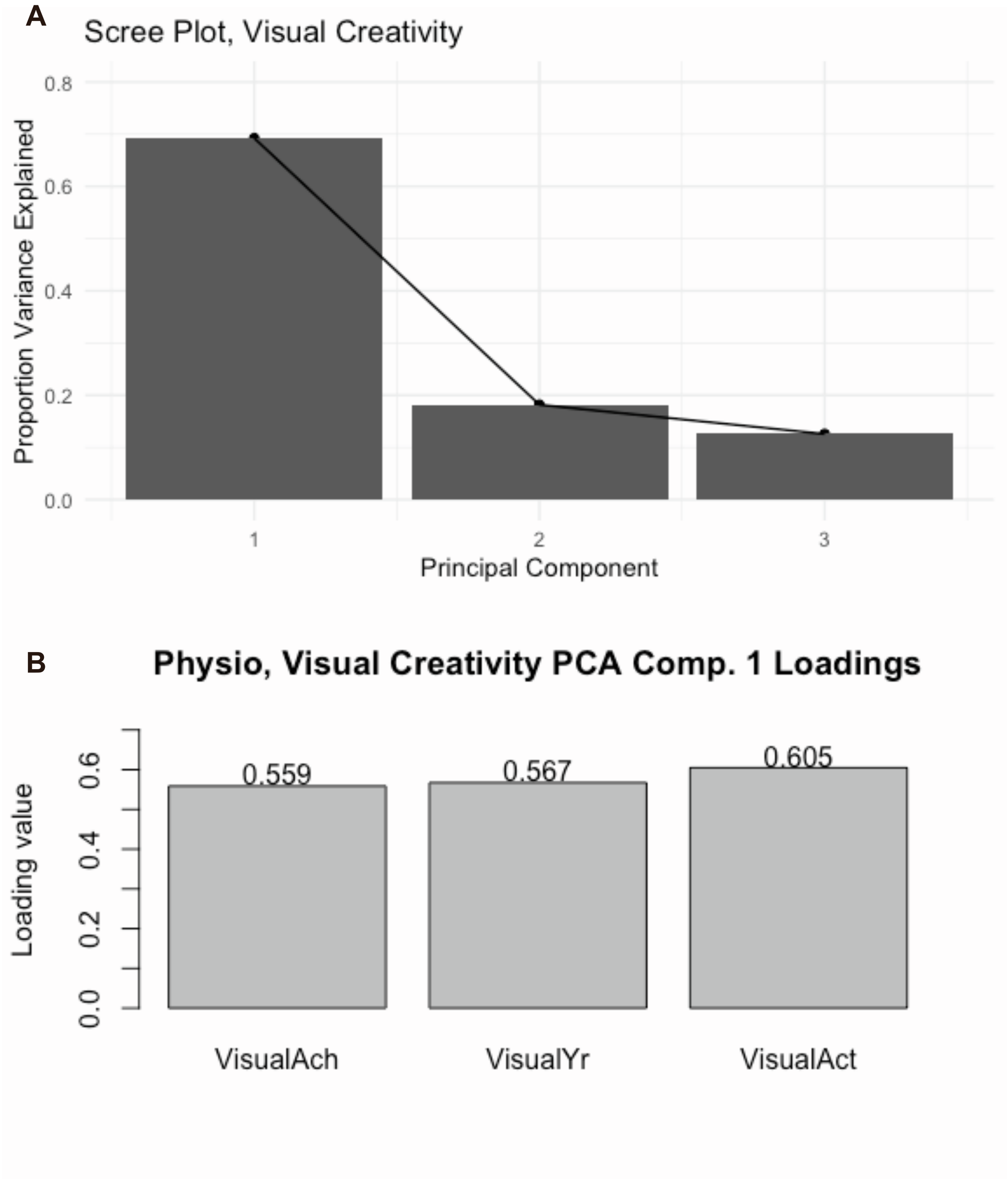

Fig. S14: Earlier PCA (Figure S9) included 99 participants. Here we show the **(A)** Scree plot and **(B)** component loadings of PCA performed on Visual Creativity variables using only the 98 participants with valid physio data. Related to STAR Methods.

Figure S15:

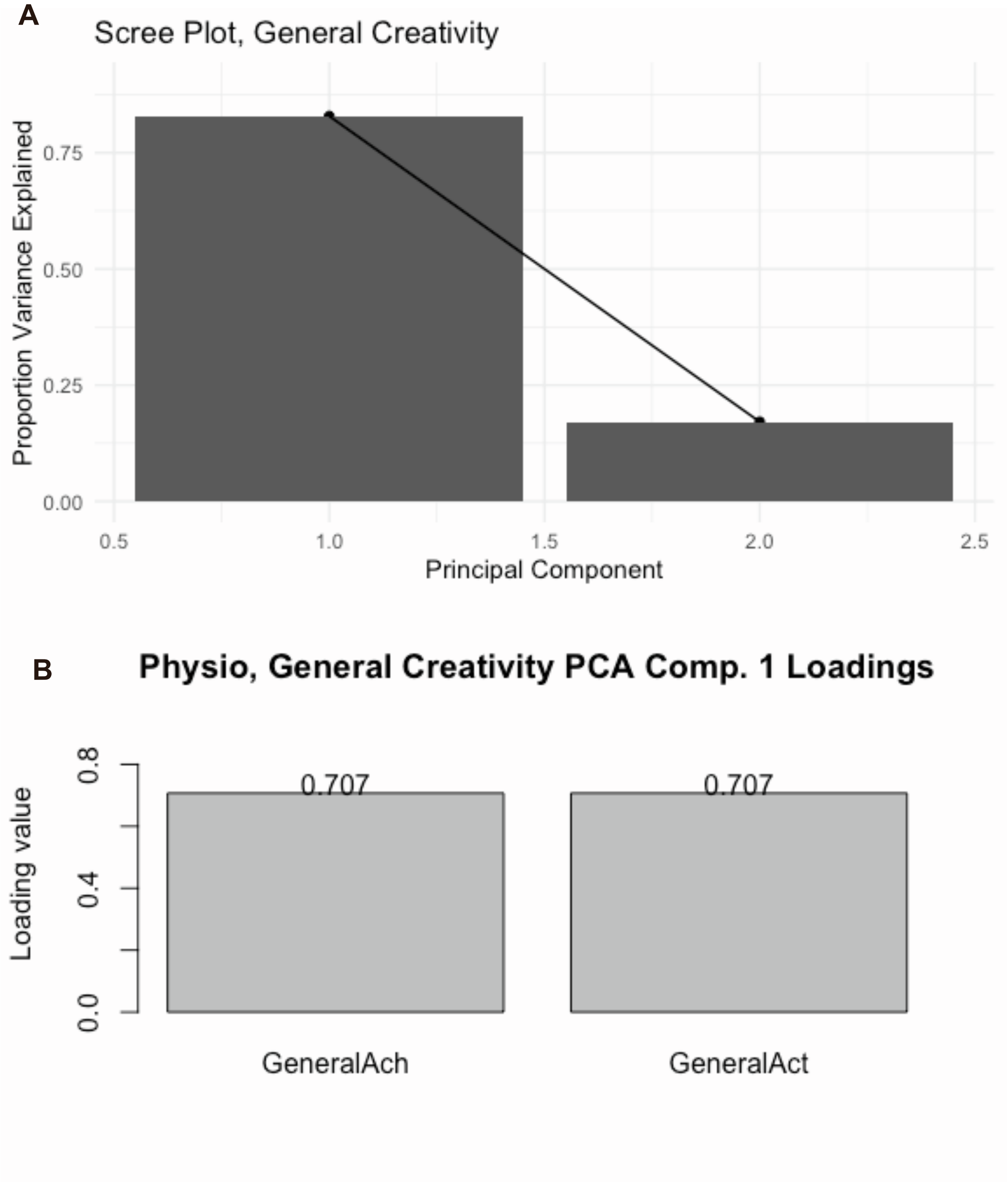

Fig. S15: Earlier PCA (Figure S9) included 99 participants. Here we show the **(A)** Scree plot and **(B)** component loadings of PCA performed on General Creativity variables using only the 98 participants with valid physio data. Related to STAR Methods.

Figure S16:

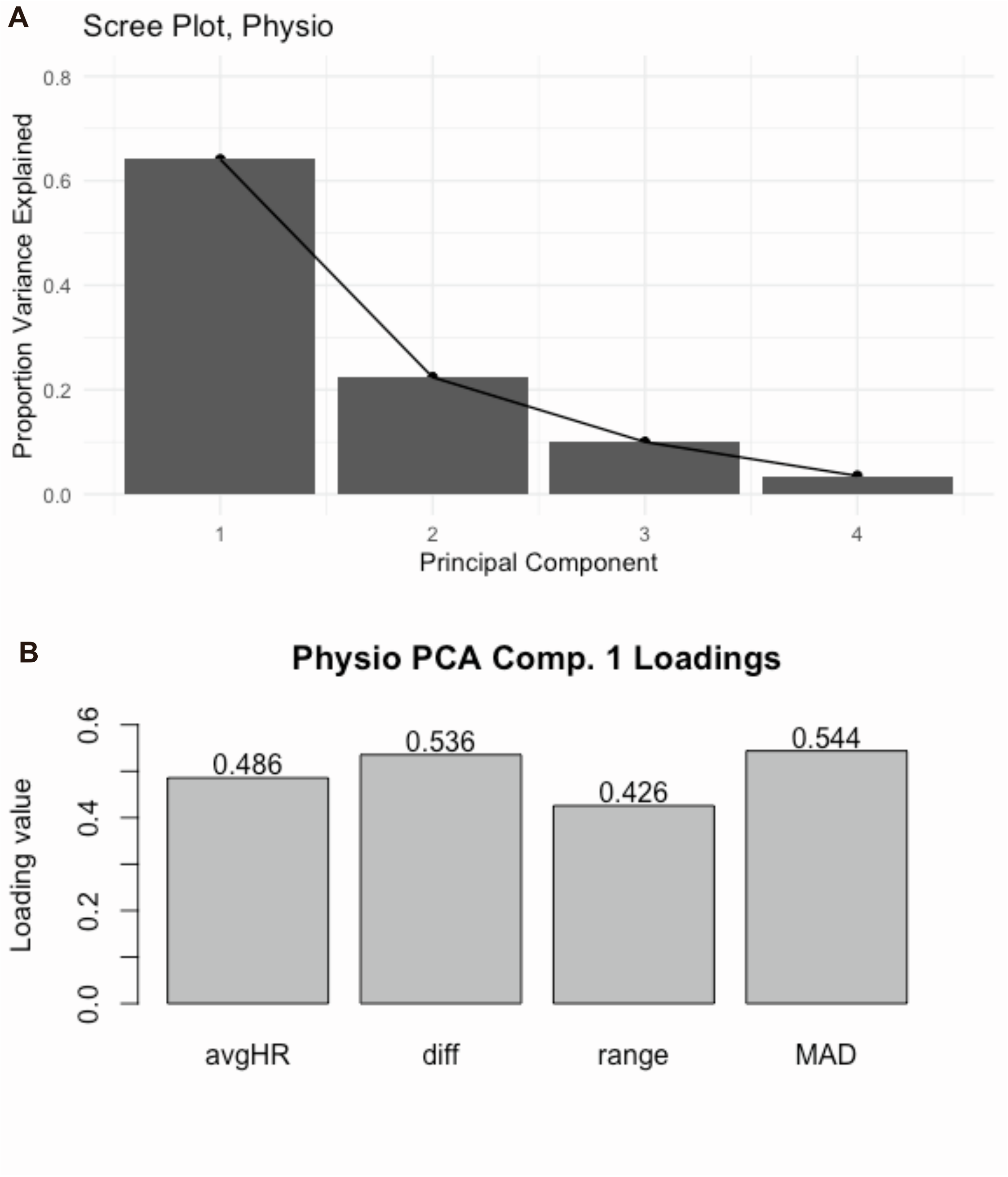

Fig. S16: (A) Scree plot and **(B)** component loadings of PCA performed on heart-rate (Physiological Reactivity) variables. Related to STAR Methods.

Data S12: Pre-registered exploratory analyses: Sleep

The following analyses were reported in our pre-registration as additional, exploratory procedures. They are not intended to be confidently interpreted, but rather to motivate future investigations in this sphere of research.

Firstly, we sought to examine if the conditions differently affected sleep quality, as measured by Richards-Campbell Sleep Questionnaire [S22] the night following each condition. While significant research has examined the relationship between sleep and creativity in the form of sleep loss's detrimental effect [S23] or the role of memory formation and creative problem-solving during sleep ([S24]; [S25]), surprisingly little research has examined if engaging in creative tasks benefits post-task sleep quality in general.

As mentioned, two participants did not report data on the third day, reducing our sample to 97 for this analysis. A 2 (Time: night after condition 1, night after condition 2) x 2 (Condition: Paint, Maze) repeated-measures ANOVA with sleep quality as the dependent variable revealed a main effect of Time, $F(1, 96) = 5.02$, $p = .027$, partialeta^2 = .050, such that sleep quality increased following each condition, $t(96) = 2.240$, $p = .027$, $b = 2.69$. No main effect of Condition was found, $F(1, 96) = 0.29$, $p = .594$, partialeta^2 = .003, nor an interaction between Time and Condition, $F(1, 96) = 0.03$, $p = .854$, partialeta^2 < .001. Thus, painting did not differently affect sleep quality than the maze control task. The main effect of condition remains when including condition order as a between-subjects variable.

Effect	df	MSE	F	pes	p.value
Condition	1, 96	146.67	0.29	.003	.594
variable	1, 96	139.80	5.02 *	.050	.027
Condition:variable	1, 96	402.76	0.03	<.001	.854

```
## variable_pairwise         estimate  SE df t.ratio p.value
## pre_sleep - nextday_sleep    -2.69 1.2 96  -2.240  0.0274
##
## Results are averaged over the levels of: Condition
```

Figure S17:

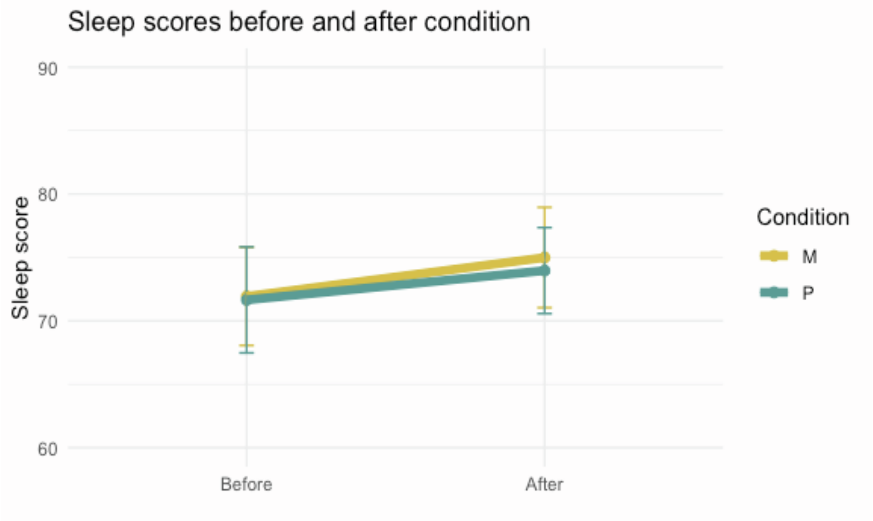

Fig. S17: Change in sleep scores between the night before and the night after each condition. There emerged a significant main effect of Time (*p* = .027) such that sleep scores increased after both conditions, but a non-significant interaction between Time and Condition. Related to Figure 1. Data are represented as mean +/- 95% confidence intervals.

Data S13: Sleep effects maintain when including Condition Order (CondOrder) as a moderator

Contrasts set to contr.sum for the following variables: CondOrder

Effect	df	MSE	F	pes	p.value
CondOrder	1, 95	782.36	0.35	.004	.556
Condition	1, 95	140.79	0.32	.003	.571
CondOrder:Condition	1, 95	140.79	5.01 *	.050	.028
variable	1, 95	140.79	5.01 *	.050	.028
CondOrder:variable	1, 95	140.79	0.32	.003	.571
Condition:variable	1, 95	404.07	0.03	<.001	.861
CondOrder:Condition:variable	1, 95	404.07	0.69	.007	.409

Data S14: Pre-registered exploratory analyses: Likelihood to engage in future creative behaviors

Additionally, we sought to examine if conditions differently affected the degree to which the individual was more likely to engage in future creative behavior outside of the experiment. Should creative engagement benefit mental health (as we found in painting for anxiety), will participants have their own agency to seek out this intervention in the future? We assessed this via a simple 1 (Extremely unlikely) - 5 (Extremely likely) Likert-scored item asking “After today, how likely would you be to engage in creative activities in the near future? This may include, for instance, going to a museum, practicing an instrument, engaging with arts & crafts, etc.” Due to participant error, answers from three participants were not collected, leading to a sample of 96 for this analysis. A paired t-test found a non-significant difference in conditions, $t(95) = 1.03$, $p = .31$. Thus, painting ($M = 4.09$, $SD = 1.09$) did not differently encourage future creative behavior than the control maze condition ($M = 4.02$, $SD = 1.03$), suggesting a role for environmental- or clinician-based encouragement for art therapy.

```
##  Paired t-test
##
## data:  paint_fca$FutureCreatAct and maze_fca$FutureCreatAct
## t = 1.0318, df = 95, p-value = 0.3048
## alternative hypothesis: true mean difference is not equal to 0
## 95 percent confidence interval:
##  -0.09626308  0.30459641
## sample estimates:
## mean difference
##       0.1041667

## [1] "Cohens D: 0.105304372686597"

## [1] "Mean FutureCreatAct, paint: 4.09183673469388"

## [1] "SD FutureCreatAct, paint: 1.08491754348541"

## [1] "Mean FutureCreatAct, maze: 4.02061855670103"

## [1] "Mean FutureCreatAct, maze: 1.0305680214015"
```

Data S15: Pre-registered exploratory analyses: Rated painting creativity

We sought to explore if the actual painting produced by the paintings carried any significant meaning as it related to the anti-anxiety effect found in the painting condition but not mazes. That is, do participants who express their thoughts more creatively onto the canvas enjoy the benefits of anxiety reduction more (or less), suggesting an expertise effect? Thus, the three most senior authors (all of whom have significant experience in arts and creativity psychology) rated the paintings for creativity in a paradigm similar to [S26]. Additionally, one of the raters is a professional painter. All 99 paintings were rated on a scale from 1 (not at all creative) to 7 (very creative). Each rater rated each painting once. All raters used the full scale.

First, we assessed the interrater reliability between the three raters using intraclass correlation (ICC). ICC was the preferred reliability measure given that we had more than two raters and over five levels on the creativity rating scale ([S27]; [S28]). A two-way random effects ICC model demonstrated poor agreement (ICC = .33) among raters. Accordingly, as in [S26] and other recent creativity-related studies (e.g., [S29]; [S30]), we implemented many-facet Rasch models (MFRM; [S31]) to correct for rater severity bias. We used the TAM package (version 4.1.4; [S32]) to carry out this MFRM procedure. MFRM results demonstrated that the three raters varied in rating severity from -0.33 (less severe, more lenient) to 0.22 (more severe, less lenient) in the Z-metric, which justified using faceted Rasch scaling. The resultant “fair average” Rasch scores (i.e., corrected painting creativity scores), when adjusting for rater severity, ranged from -1.08 to 1.29. The final EAP reliability was .63, demonstrating a meaningful procedure for correcting for rater bias.

```
## Call: ICC(x = ratings[, 1:3])
##
## Intraclass correlation coefficients
##                          type  ICC   F df1 df2       p lower bound upper bound
## Single_raters_absolute   ICC1 0.31 2.3  98 198 2.0e-07        0.19        0.44
## Single_random_raters     ICC2 0.33 2.6  98 196 4.1e-09        0.20        0.46
## Single_fixed_raters      ICC3 0.35 2.6  98 196 4.1e-09        0.23        0.48
## Average_raters_absolute ICC1k 0.57 2.3  98 198 2.0e-07        0.41        0.70
## Average_random_raters   ICC2k 0.59 2.6  98 196 4.1e-09        0.43        0.72
## Average_fixed_raters    ICC3k 0.62 2.6  98 196 4.1e-09        0.47        0.73
##
##  Number of subjects = 99     Number of Judges =  3
## See the help file for a discussion of the other 4 McGraw and Wong estimates,

## EAP Reliability

## 0.625

## Rater Severity

## -0.33 0.108 0.223

## Range of Scores

## -1.082 1.292
```

These new participant painting creativity scores were included as a centered, between-subjects variable in the original 2 (Condition: Paint, Maze) x 2 (Time: pre, post) ANOVA with STAI scores as the dependent variable. We sought to examine if there was a significant moderation of the original Condition x Time interaction based on the painting creativity scores. The ANOVA demonstrated that the original Condition x Time interaction remained $F(1, 97) = 21.37$, $p = .019$, partialeta^2 = .056, with a non-significant 3-way interaction between painting creativity scores, Condition, and Time ($F(1, 97) = 0.02$, $p = .876$, partialeta^2 < .001). Thus, the rated creativity of the painting product itself did not meaningfully moderate the ability to derive anti-anxiety effects from painting specifically (as compared to the control maze condition). To support this claim, we also ran a correlation test between the STAI difference (post-pre) scores with the painting creativity scores and found a non-significant correlation of $r(97) = .02$, $p = .88$.

Effect	df	MSE	F	pes	p.value
centered_Creatratings	1, 97	322.30	0.21	.002	.644
Condition	1, 97	72.07	4.00 *	.040	.048
centered_Creatratings:Condition	1, 97	72.07	2.06	.021	.155
variable	1, 97	36.45	38.14 ***	.282	<.001
centered_Creatratings:variable	1, 97	36.45	0.00	<.001	.949
Condition:variable	1, 97	21.37	5.72 *	.056	.019
centered_Creatratings:Condition:variable	1, 97	21.37	0.02	<.001	.876

```
##  Pearson's product-moment correlation
##
## data:  (paint$post_STAI - paint$pre_STAI) and mfrm_ratings$EAP
## t = 0.15409, df = 97, p-value = 0.8779
## alternative hypothesis: true correlation is not equal to 0
## 95 percent confidence interval:
##  -0.1823312  0.2123996
## sample estimates:
##        cor
## 0.01564372
```

Data S16: Pre-registered exploratory analyses: Flow state correlates

Lastly, we examined various relationships between total flow state (and its nine sub-scales) during painting and other potentially relevant measures including physiological reactivity, perceived self-performance, and the creative quality of the actual produced paintings. We chose these measures for several reasons. Firstly, research suggests that flow involves intense attentional processes, which can alter physiological processes like respiration, blood pressure, and other cardiovascular systems [S33]. Thus, we sought to examine how flow related to our measure of physiological reactivity as measured by our PCA physiology primary component. Next, there is a rich literature investigating the relationship between flow and task performance, particularly in sports. Indeed, a positive relationship seems to exist between the two [S34]. However, flow is also phenomenologically associated with a lack of self-directed cognition (i.e., "the disappearance of the self-consciousness"; [S35], p. 757), thought to be a consequence of neural patterns of hypofrontality [S35]. Thus, the degree to which an individual is able to introspect (and thus bring the self into consciousness) on his/her performance and how this relates to reported flow is an interesting and perhaps paradoxical question that we wished to explore. Lastly, and closely related, it is well-known that flow is involved the cognitive processes underlying immersive artistic tasks; accordingly, extending these measures to the creative product generated from these processes is another interesting and open question.

To accomplish this analysis, we performed targeted, zero-order Pearson's correlations among these measures (Table S1). To summarize, we see evidence of significant and moderate-to-high positive relationships among nearly all facets of flow state with perceived self-performance, suggesting that the two constructs capture a similar latent state of accomplishment. In addition, some, but not many, relationships emerged among Physiological Reactivity (the aggregated physiological variable) and subscores of flow state. In particular, physiological reactivity significantly positively correlated with Flow State: Clear Goals, Flow State: Concentration, and Flow State: Autotelic. Lastly, we found that the MFRM-corrected scores of painting creativity ratings significantly positively associated with Flow State: Challenge and Flow State: Autotelic, perhaps suggesting a route by which more-creative products can emerge through cognitive processes of flow.

Table S1

	**SelfPeformance**	**Physio Reactivity**	**Painting Creativity**
**FS: Challenge**	**0.54***	0.12	**0.23***
**FS: Action-Awareness**	**0.36***	0.15	0.11
**FS: Clear Goals**	**0.40***	**0.21***	0.03
**FS: Unambiguous Feedback**	**0.26***	0.14	0.07
**FS: Concentration**	**0.43***	**0.26***	0.18
**FS: Paradox of Control**	**0.32****	0.05	0.11
**FS: Loss of Self Consciousness**	**0.48***	0.01	0.11
**FS: Time Transformation**	-0.20	-0.04	0.03
**FS: Autotelic**	**0.62***	**0.25***	**0.24***
**FS: Total**	**0.55***	0.20	0.19

Table S1: Correlations between flow state (FS) scores and theoretically relevant measures during painting. No corrections were performed as we do not formally interpret these findings but only illustrate potential relationships meant to motivate future research. ***$p < .001$, **$p < .01$. *$p < .05$

Data S17: Openness to Experience as a moderator of Physiological Reactivity (reviewer request)

Effect	df	MSE	F	pes	p.value
centered_open	1, 94	3.06	1.48	.016	.226
centered_maas	1, 94	3.06	0.06	<.001	.814
centered_open:centered_maas	1, 94	3.06	0.47	.005	.497
Condition	1, 94	1.78	13.37 ***	.125	<.001
centered_open:Condition	1, 94	1.78	6.00 *	.060	.016
centered_maas:Condition	1, 94	1.78	0.34	.004	.561
centered_open:centered_maas:Condition	1, 94	1.78	0.19	.002	.665

Figure S18:

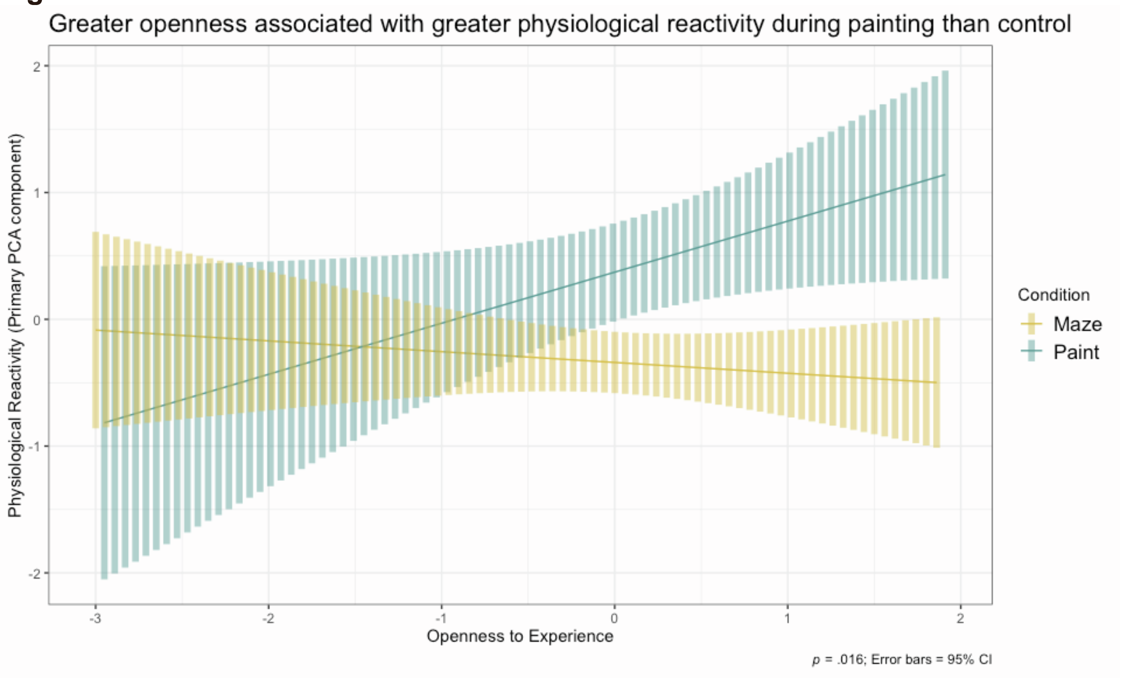

Fig. S18: As individual Openness to experience increased, participants demonstrated greater physiological reactivity in painting compared to mazes, *p* = .016, η_p^2 = .060. This mirrors the effect of General Creativity. Related to Figure 5. Plot depicts regressions based on individual data points +/- 95% confidence intervals.

Data S18: Openness to Experience, Mindfulness as non-significant moderators of Primary (STAI) ANOVA (reviewer request)

Effect	df	MSE	F	pes	p.value
centered_maas	1, 95	295.44	7.11 **	.070	.009
centered_open	1, 95	295.44	0.03	<.001	.864
centered_maas:centered_open	1, 95	295.44	1.11	.012	.294
Condition	1, 95	71.68	3.01 +	.031	.086
centered_maas:Condition	1, 95	71.68	3.00 +	.031	.087
centered_open:Condition	1, 95	71.68	0.13	.001	.718
centered_maas:centered_open:Condition	1, 95	71.68	0.61	.006	.438
variable	1, 95	35.46	32.47 ***	.255	<.001
centered_maas:variable	1, 95	35.46	2.89 +	.030	.092
centered_open:variable	1, 95	35.46	2.16	.022	.145
centered_maas:centered_open:variable	1, 95	35.46	1.82	.019	.181
Condition:variable	1, 95	21.64	5.96 *	.059	.016
centered_maas:Condition:variable	1, 95	21.64	0.04	<.001	.843
centered_open:Condition:variable	1, 95	21.64	0.56	.006	.456
centered_maas:centered_open:Condition:variable	1, 95	21.64	0.32	.003	.573

References

[S1] Brown, K.W. & Ryan, R.M. (2003). The benefits of being present: Mindfulness and its role in psychological well-being. Journal of Personality and Social Psychology, 84, 822-848.

[S2] McCrae, R. R. (1987). Creativity, divergent thinking, and openness to experience. *Journal of Personality and Social Psychology*, *52*(6), 1258–1265. https://doi.org/10.1037/0022-3514.52.6.1258

[S3] Costa, P. T., Jr., & McCrae, R. R. (1992). Revised NEO Personality Inventory (NEO-PI-R) and NEO Five-Factor Inventory (NEO-FFI) professional manual. Odessa, FL: Psychological Assessment Resources.

[S4] Cattell, R. B., & Cattell, A. K. (1973). Measuring intelligence with the culture fair tests. Champaign, IL: Institute for Personality and Ability Testing.

[S5] Diedrich, J., Jauk, E., Silvia, P. J., Gredlein, J. M., Neubauer, A. C., & Benedek, M. (2018). Assessment of real-life creativity: The Inventory of Creative Activities and Achievements (ICAA). *Psychology of Aesthetics, Creativity, and the Arts*, *12*(3), 304–316. https://doi.org/10.1037/aca0000137

[S6] Karwowski, M. (2014). Creative mindsets: Measurement, correlates, consequences. Psychology of Aesthetics, Creativity, and the Arts, 8(1), 62–70. doi:10.1037/a0034898

[S7] Spielberger, C. D. (1983). *State-Trait Anxiety Inventory for Adults* [Dataset]. https://doi.org/10.1037/t06496-000

[S8] Carriere, J. S. A., Seli, P., & Smilek, D. (2013). Wandering in both mind and body: Individual differences in mind wandering and inattention predict fidgeting. Canadian Journal of Experimental Psychology / Revue canadienne de psychologie expérimentale, 67(1), 19–31. doi:10.1037/a0031438

[S9] Gross, J. J., & John, O. P. (2003). Individual differences in two emotion regulation processes: Implications for affect, relationships, and well-being. Journal of Personality and Social Psychology, 85, 348–362. https://doi.org/10.1037/0022-3514.85.2.348

[S10] Lovibond, S. H., & Lovibond, P. F. (1995). *Depression Anxiety Stress Scales (DASS--21, DASS--42)* [Database record]. APA PsycTests. doi:10.1037/t01004-000

[S11] Jackson, S. A., & Marsh, H. W. (1996). Development and Validation of a Scale to Measure Optimal Experience: The Flow State Scale. Journal of Sport and Exercise Psychology, 18(1), 17–35. https://doi.org/10.1123/jsep.18.1.17

[S12] Nissen, M., Slim, S., Jäger, K., Flaucher, M., Huebner, H., Danzberger, N., Fasching, P. A., Beckmann, M. W., Gradl, S., & Eskofier, B. M. (2022). Heart Rate Measurement Accuracy of Fitbit Charge 4 and Samsung Galaxy Watch Active2: Device Evaluation Study. JMIR Formative Research, 6(3), e33635. https://doi.org/10.2196/33635

[S13] Jachymek, M., Jachymek, M. T., Kiedrowicz, R. M., Kaźmierczak, J., Płońska-Gościniak, E., & Peregud-Pogorzelska, M. (2022). Wristbands in Home-Based Rehabilitation—Validation of Heart Rate Measurement. Sensors, 22(1), Article 1. https://doi.org/10.3390/s22010060

[S14] Brydges, C. R. (2019). Effect Size Guidelines, Sample Size Calculations, and Statistical Power in Gerontology. Innovation in Aging, 3(4), igz036. https://doi.org/10.1093/geroni/igz036

[S15] Hermans, F., Blondeel, A., Arents, E., Calders, P., Troosters, T., Derom, E., & Demeyer, H. (2023). Validity of the Fitbit Charge 4 to measure daily steps, oxygen saturation and resting heart rate in patients with COPD. European Respiratory Journal, 62(suppl 67). https://doi.org/10.1183/13993003.congress-2023.PA361

[S16] Neacsiu, A. D., Beynel, L., Powers, J. P., Szabo, S. T., Appelbaum, L. G., Lisanby, S. H., & LaBar, K. S. (2022). Enhancing Cognitive Restructuring with Concurrent Repetitive Transcranial Magnetic

Stimulation: A Transdiagnostic Randomized Controlled Trial. Psychotherapy and Psychosomatics, 91(2), 94–106. https://doi.org/10.1159/000518957

[S17] Appelhans, B. M., & Luecken, L. J. (2006). Heart Rate Variability as an Index of Regulated Emotional Responding. Review of General Psychology, 10(3), 229–240. https://doi.org/10.1037/1089-2680.10.3.229

[S18] Kazmi, S. Z. H., Zhang, H., Aziz, W., Monfredi, O., Abbas, S. A., Shah, S. A., Kazmi, S. S. H., & Butt, W. H. (2016). Inverse Correlation between Heart Rate Variability and Heart Rate Demonstrated by Linear and Nonlinear Analysis. PLOS ONE, 11(6), e0157557. https://doi.org/10.1371/journal.pone.0157557

[S19] Ungi, I., Thury, A., & Csanady, M. (1995). Investigation of the correlation between heart rate and heart rate variability. Computers in Cardiology 1995, 189–191. https://doi.org/10.1109/CIC.1995.482604

[S20] Brouwer, A.-M., van Dam, E., van Erp, J. B. F., Spangler, D. P., & Brooks, J. R. (2018). Improving Real-Life Estimates of Emotion Based on Heart Rate: A Perspective on Taking Metabolic Heart Rate Into Account. Frontiers in Human Neuroscience, 12. https://www.frontiersin.org/articles/10.3389/fnhum.2018.00284

[S21] Montoya, Amanda K., and Andrew F. Hayes. “Two-Condition within-Participant Statistical Mediation Analysis: A Path-Analytic Framework.” Psychological Methods 22, no. 1 (2017): 6–27. https://doi.org/10.1037/met0000086.

[S22] Richards, K. C., O’Sullivan, P. S., & Phillips, R. L. (2000). Measurement of Sleep in Critically Ill Patients. Journal of Nursing Measurement, 8(2), 131–144. https://doi.org/10.1891/1061-3749.8.2.131

[S23] Horne, J. A. (1988). Sleep Loss and “Divergent” Thinking Ability. Sleep, 11(6), 528–536. https://doi.org/10.1093/sleep/11.6.528

[S24] Drago, V., Aricò, D., Heilman, K., Foster, P., Williamson, J., Montagna, P., & Ferri, R. (2010). The Correlation between Sleep and Creativity. Nature Precedings. https://doi.org/10.1038/npre.2010.4266.1

[S25] Ritter, S. M., Strick, M., Bos, M. W., Van Baaren, R. B., & Dijksterhuis, A. (2012). Good morning creativity: Task reactivation during sleep enhances beneficial effect of sleep on creative performance. Journal of Sleep Research, 21(6), 643–647. https://doi.org/10.1111/j.1365-2869.2012.01006.

[S26] Bellaiche, L., Smith, A. P., Barr, N., Christensen, A., Williams, C., Ragnhildstveit, A., Schooler, J., Beaty, R., Chatterjee, A., & Seli, P. (2023). Back to the basics: Abstract painting as an index of creativity. Creativity Research Journal, 0(0), 1–16. https://doi.org/10.1080/10400419.2023.2243100

[S27] Bajpai, S., Bajpai, R., & Chaturvedi, H. (2015). Evaluation of Inter-Rater Agreement and Inter-Rater Reliability for Observational Data: An Overview of Concepts and Methods. Journal of the Indian Academy of Applied Psychology, 41, 20–27.

[S28] Hallgren, K. A. (2012). Computing Inter-Rater Reliability for Observational Data: An Overview and Tutorial. Tutorials in Quantitative Methods for Psychology, 8(1), 23–34.

[S29] Primi, R., Silvia, P. J., Jauk, E., & Benedek, M. (2019). Applying many-facet Rasch modeling in the assessment of creativity. Psychology of Aesthetics, Creativity, and the Arts, 13(2), 176–186. https://doi.org/10.1037/aca0000230

[S30] Silvia, P. J., Christensen, A. P., & Cotter, K. N. (2021). Right-wing authoritarians aren’t very funny: RWA, personality, and creative humor production. Personality and Individual Differences, 170, 110421. https://doi.org/10.1016/j.paid.2020.110421

[S31] Linacre, J. M. (1994). Many-faceted Rasch measurement (2nd ed.). Chicago, IL: University of Chicago.

[S32] Robitzsch, A., Kiefer, T., & Wu, M. (2022). TAM: Test Analysis Modules (4.1-4) [Computer software]. https://cran.r-project.org/web/packages/TAM/index.html

[S33] Ullén, F., de Manzano, Ö., Theorell, T., & Harmat, L. (2010). The Physiology of Effortless Attention: Correlates of State Flow and Flow Proneness (pp. 205–218). https://doi.org/10.7551/mitpress/9780262013840.003.0011

[S34] Harris, D. J., Allen, K. L., Vine, S. J., & Wilson, M. R. (2023). A systematic review and meta-analysis of the relationship between flow states and performance. International Review of Sport and Exercise Psychology, 16(1), 693–721. https://doi.org/10.1080/1750984X.2021.1929402

[S35] Dietrich, A. (2004). Neurocognitive mechanisms underlying the experience of flow. Consciousness and Cognition, 13(4), 746–761. https://doi.org/10.1016/j.concog.2004.07.002
